# Enriched Molecular-Level View of Saline Wetland Soil Carbon by Sensitivity-Enhanced Solid-State NMR

**DOI:** 10.1021/jacs.4c11830

**Published:** 2024-12-19

**Authors:** Wancheng Zhao, Elizabeth C. Thomas, Debkumar Debnath, Faith J. Scott, Frederic Mentink-Vigier, John R. White, Robert L. Cook, Tuo Wang

**Affiliations:** † Department of Chemistry, Michigan State University, East Lansing, Michigan 48824, United States; ‡ Department of Chemistry, Louisiana State University, Baton Rouge, Louisiana 70803, United States; § National High Magnetic Field Laboratory, Florida State University, Tallahassee, Florida 23310, United States; ∥ Department of Oceanography & Coastal Sciences, Louisiana State University, Baton Rouge, Louisiana 70803, United States; ⊥ Coastal Studies Institute, Louisiana State University, Baton Rouge, Louisiana 70803, United States

## Abstract

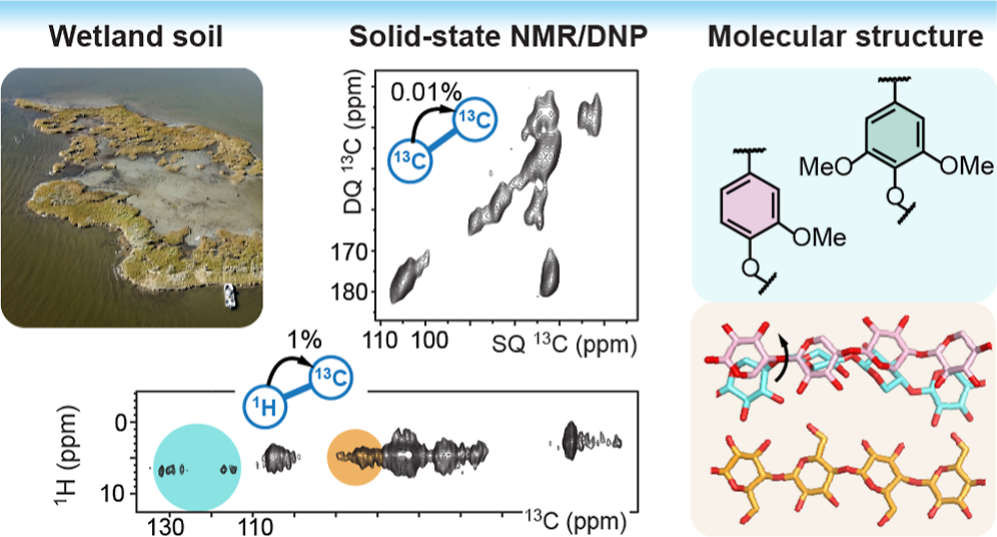

Soil organic matter (SOM) plays a major role in mitigating greenhouse gas emission and regulating earth’s climate, carbon cycle, and biodiversity. Wetland soils account for one-third of all SOM; however, globally, coastal wetland soils are eroding faster due to increasing sea-level rise. Our understanding of carbon sequestration dynamics in wetlands lags behind that of upland soils. Here, we employ solid-state nuclear magnetic resonance (ssNMR) to investigate the molecular-level structure of biopolymers in wetland soils spanning 11 centuries. High-resolution multidimensional spectra, enabled by dynamic nuclear polarization (DNP), demonstrate enduring preservation of molecular structures within herbaceous plant cores, notably condensing aromatic motifs and carbohydrates, even over a millennium, with the preserved cores constituting a decreasing minority among molecules from decomposition and repolymerization with depth and age. Such preserved cores occur alongside molecules from the decomposition of loosely packed parent biopolymers. These findings emphasize the relative vulnerability of coastal wetland SOM when exposed to oxygenated water due to geological and anthropogenic changes.

## Introduction

Soil organic matter (SOM) and associated soil organic carbon (SOC) critically represents one of the major reservoirs of carbon on the planet and is critical to ecosystem services, from the molecular to the global scale.^1,2^ Just the top 1 m of the world’s soil contains 1500 petagrams (Pg, billion tons) of carbon, approximately twice the carbon pool contained in the entire atmosphere^3^ and more than the atmosphere and vegetation combined.^4^ Soil has been a sink to about 210 ± 45 Pg of carbon between the years 1850 and 2021, mitigating around 100 ppm of atmospheric CO_2_ levels.^5^ Wetland soils contain approximately one-third of this SOC despite occupying only 5–6% of the earth’s land surface area.^6^ Coastal wetlands, known as the blue carbon ecosystems, occupy only 0.07–0.22% of the earth’s surface but sequester 0.08–0.22 Pg carbon each year, accounting for more than half of the carbon buried in the oceans annually,^7−9^ and store the vast majority of this carbon for hundreds to thousands of years.

The anaerobic conditions of wetland soil cause a reduced metabolic efficiency of microbes in acquiring energy during decomposition of complex organic carbon compounds, which leads to increased preservation of carbon in the soil profile unless hydrologic conditions change.^10^ Additionally, for coastal wetlands, methane emissions are minimal due to a relatively high poised redox potential^11^ but are projected to rise significantly due to climate warming.^12^ Subsidence, sea-level rise, and wave energy promote wetland soil collapse into the surrounding shallow, estuarine aerobic water column, resulting in the oxidation of SOM and release of the stored carbon back to the atmosphere as primarily carbon dioxide.^13−15^ There is a critical need to understand, model, and accurately predict the dynamics of SOM, and hence SOC, in a scalable and widely applicable manner.^16^ Thus, detailed molecular information, including functionality, isomerism, and conformations of the sequestered carbon pool, is a fundamental requirement. Currently, there is limited work on this advanced level of carbon sequestration within coastal wetlands, as most molecular characterization work has been focused on upland soils (Supporting Information).

The persistence of SOC was considered to occur through the formation of recalcitrant macromolecules by polymerization during a process called humification.^17^ This decomposition and repolymerization (humification) process consists of (1) the transformation of parent organic inputs, e.g., plant residues, through biotic and abiotic processes in which aromatic moieties are favored due to stability considerations, followed by (2) the recombination of these transformed products into larger recalcitrant molecules, in humic materials, such as polyphenols. Presently, the dynamic stability concept prevails, whereby SOC is preserved as an organic entity via molecular preservation for periods of time, in concert with dynamically changing organic carbon speciation.^18^ This new view also breaks SOM down into two major pools: particulate organic matter (POM), which includes occluded SOM and plant materials, and mineral-associated organic matter (MAOM), which contains mostly small organic molecules and biological metabolites.^19,20^ Over time, these two more recalcitrant pools may become accessible and therefore can be viewed as shorter-term stable carbon deposits in the context of geologic time.^21^ Most research on this new paradigm was focused on upland or mineral soils, with the MAOM fraction receiving the bulk of the attention, although the focus is beginning to shift.^20,22,23^ Most coastal wetlands are POM-dominated systems with a high organic matter/mineral matter ratio. Globally, per unit area, coastal wetlands are considered the most carbon-rich dynamic reservoirs of organic carbon, warranting a closer examination of this critical carbon pool most vulnerable to loss as the sea level continues to rise.^24,25^ In order to predict the fate of these carbon stores, it is essential to understand the carbon dynamics in these wetlands under increasing sea-level rise, which shows no signs of deceleration. As sea levels rise, the substantial carbon stores within global wetlands are at risk, with world’s stable coastlines beginning to catch up to the high wetland loss rates observed in the Louisiana Delta.^26^ The loss of such carbon reserves on a global scale has the potential to offset reductions in anthropogenic carbon emissions, significantly impacting the atmospheric carbon pool.^27^

Here, we examine coastal wetland soil samples from the Mississippi River Delta, USA, that have been deposited and preserved over the past 11 centuries but whose soil C stores are being lost at an accelerated rate due to the high relative sea-level rise rate.^28,29^ Sensitivity-enhanced solid-state nuclear magnetic resonance (ssNMR) is applied, for the first time, to whole soil samples to enable rapid acquisition of 2D ^13^C/^1^H–^13^C correlation spectra, providing atomic-level structural information on the carbon fraction of SOC. This method allows for a detailed characterization of soil on the fine granular and molecular level, beyond the moiety characterization level (Supporting Information), which reveals that (1) in the initial stages of SOC sequestration, core structures of the parent herbaceous plant materials are preserved, but the aromatic and carbohydrates motifs become tightly packed, with noncarbohydrate components being concentrated in the soil, (2) in parallel, molecular decomposition and repolymerization (humification) are at play, resulting in the formation of new molecules from the decomposition of loosely packed parent biopolymers and biogeochemical processing, adding to the diversity of SOC chemical nature, (3) some structural cores of herbaceous plant biopolymers survive anaerobic microbial degradation, with their original structure and physical packing preserved up to 1000 years since deposition, and (4) changes in the carbon speciation during the sequestration processes are driven by both natural geological changes (e.g., delta lobe switching) as well as anthropogenic changes (e.g., river levees). Besides these multifaceted conceptual advances, this high-resolution and rapid technological platform also opens a new research avenue for SOC analysis in whole soils.

## Materials and Methods

### Collection of Soil Material

Soil cores (2 m in length) were extracted with a polycarbonate core tube from a brackish *Spartina alterniflora*-dominated island in the Barataria Basin, Louisiana, USA (GPS coordinates: 29.44358, −89.899722). Two cores were extracted at different distances (1 and 2 m, respectively) from the shoreline of the island. The extracted materials were divided into 10 cm sections based on depth, stored on ice during transportation, and then kept at 4 °C for storage until analyzed.

### Hydrofluoric Acid Treatment

Visible plant matter was removed from the dried soil samples. Each sample was ground with a mortar and pestle set until the material could pass through a 125 μm sieve. Around 600 mg of ground material was transferred into a 15 mL centrifuge tube, and 10 mL of a 2% HF solution was added. The tube was capped and turned end-over-end in a rotary mixer throughout for 9 different time intervals in the following sequence: five 1 h intervals, then a 16 h interval followed by two 24 intervals, and finally, a 72 h interval. In between these intervals, the tubes were placed into a benchtop centrifuge and spun at 2000 rpm for 20 min at room temperature. After centrifugation, the 2% HF solution was decanted and replaced with a freshly prepared 2% HF solution. The soil samples were then vacuum-filtered with 18 MΩ water three times to remove the excess HF and freeze-dried for 24 h. This protocol was modified from a previously reported method.^30^

### Solid-State NMR Spectroscopy

For each soil sample, 95–105 mg of HF-treated material was packed into a 4 mm zirconia rotor and measured on a Bruker Avance 400 MHz (9.4 T) NMR spectrometer. Most experiments were conducted using a 4 mm probe under 14 kHz MAS at 298 K. 1D semiquantitative spectra were measured using the multiCP pulse sequence,^31^ with 11 CP blocks applied. Each CP block used a 1.1 ms contact time, with a delay of 0.6 s between blocks. The acquisition time was set to 25 ms, and the recycling delay was 1 s. For each sample, 16,384 scans were recorded within 35 h. The field strengths of the radiofrequency pulses were 71.4 kHz for both ^13^C and ^1^H hard pulses and 62.5 kHz for ^1^H decoupling. The ^13^C chemical shifts were externally referenced to the tetramethylsilane scale by calibrating the adamantane CH_2_ peak to 38.48 ppm. In this work, all ssNMR and dynamic nuclear polarization (DNP) spectra were collected using the software Topspin 4.0 and analyzed in the Topspin 4.1 version. Graphs were plotted using OriginPro 2019b software and Adobe Illustrator CC Cs6 V16.0.0.

To analyze the content of different carbon pools, deconvolution was performed on the 1D semiquantitative multiCP ^13^C spectra using DMfit^32^ following the positions of the peaks resolved from 2D DNP spectra, as detailed in Table S1. The intensity was further calibrated by the intensity ratios between multiCP spectra and the quantitative direct polarization (DP) spectra measured with long recycle delays of 90 s (Table S2). This allowed us to convert peak intensities into carbon percentages for different structural motifs, including carbohydrates, aromatic, carbonyl, and aliphatic components, as well as the ratios of different carbon sites within each category (Table S3).

1D rotor-synchronized nonquaternary suppression (NQS) spectra were collected under 14 kHz to identify quaternary carbons.^33,34^ Signals from the protonated carbons were dephased using two delays (30 μs × 2) without heteronuclear decoupling. The CP contact time was 2 ms. The acquisition time and the recycle delay were set to 41 ms and 2 s, respectively. In addition, 1D conventional ^13^C CP spectra were collected to compare with NQS spectra, with identical experimental parameters.

Quantitative DP experiment was also conducted using a 90 s recycle delay on soil sample 7. The acquisition time was set to 25 ms; 6144 scans were recorded within 154 h. The MAS frequency was 14 kHz. The same quantitative DP experiment was also conducted on an empty rotor using a 10 s recycle delay. The DP experiments were conducted by using a 3.2 mm HCN probe on a Bruker Avance Neo 400 MHz (9.4 T) NMR spectrometer. The quantitative DP spectrum, following subtraction of the rotor signal, serves as the reference standard for calibrating carbon signal intensities within multiCP spectra. Normalization to the dominant aromatic peak yields scaling factors for specific carbon chemical shift regions in the multiCP spectra: 1.0 for the 180–165 ppm range, 1.15 for 165–145 ppm, 1.0 for 145–100 ppm, 0.47 for 100–65 ppm, 0.59 for 65–50 ppm, and 0.57 for 50–0 ppm. These scaling factors were then applied to the integrated data from spectral deconvolution, enabling accurate intensity calibration across different carbon structural motifs (Table S2).

### Preparation of Soil and Plant Samples for MAS-DNP

A stock solution, which is often referred as the DNP juice, was prepared using a mixture of D_2_O and H_2_O (90:10 Vol %) and 10 mM AsymPolPOK biradical (Catalogue# C015P01, CortecNet).^35^ Another two stock solutions were also prepared with the same radical concentration but using different solvents of DMSO-*d*_6_/D_2_O/H_2_O (10/80/10 vol %) and DMSO-*d*_6_/H_2_O (90/10 vol %). D_2_O (Catalogue# DLM-4DR-PK) and DMSO-*d*_6_ (Catalogue# DLM-10TC-PK) were from Cambridge Isotope Laboratories. The details of parameters of DNP juice composition used for each sample and the experimental parameters are listed in Table S4.

The stock solutions were mixed with three types of materials, including HF-treated and nontreated soil as well as plant materials. Around 50 mg of HF-treated soil material was impregnated in 150 μL of the stock solution and vortexed briefly. The mixture was ground mildly for 20 min using a mortar and pestle to allow the radicals to penetrate the porous components of the soil; 30 mg of the final material was then packed into a 3.2 mm sapphire rotor for measurement. For comparison, the two plant samples (*S. alterniflora*) collected from the edge of the island (on top of the soil extraction site) and 30 m inland were also processed for MAS-DNP measurement. Around 30 mg of each plant sample was subjected to the same protocol described above to mix with 10 mM AsymPolPOK. For nontreated soil samples, the protocol was modified regarding the concentration of the biradical, which has increased to 30 mM to gain more enhancement. The DNP enhancements and electron paramagnetic resonance (EPR) spectra (EMX Nano benchtop EPR) measured on the plant and soil samples were recorded. The EPR spectra were plotted by MATLAB R2020a with the toolbox EasySpin (6.0.0). The evaluation of the inhomogeneity of DNP enhancement across different molecules in the sample is explained in Supporting Information.

### 2D ^13^C/^1^H–^13^C Correlation Experiments by MAS-DNP

In unlabeled samples, the natural abundance of the ^13^C isotope is very low (1.1%), and the probability of observing connectivity between two carbon-13 nuclei in a 2D ^13^C–^13^C correlation spectrum is inhibitory (0.01%). To obtain sufficient sensitivity for measuring 2D correlation experiments,^36^ the soil and plant samples were measured on a Bruker 600 MHz/395 GHz MAS-DNP system at the National High Magnetic Field Laboratory, with the microwave irradiation power set to 12 W. The sample temperature was 104 and 100 K when the microwave was on and off, respectively. The DNP buildup time was 1.3–4.5 s for all of the MAS-DNP samples, including the HF-treated and untreated soil samples as well as the plant materials collected 30 m inland and at the edge of the island. Recycle delays were typically set to be 1.3-fold of the DNP buildup time constant for each sample. 1D ^13^C CP experiments were measured with and without microwave irradiation under 8 kHz for soil and 10.5 kHz for plant samples, with the CP contact time set to 1 ms. The experimental parameters for all 1D and 2D NMR and MAS-DNP experiments are documented in Table S4.

2D ^1^H–^13^C HETCOR experiments were carried out under 8 or 10.5 kHz MAS frequencies. ^1^H–^1^H homonuclear decoupling was achieved using either the phase-modulated Lee–Goldburg^37^ or frequency-switched Lee–Goldburg sequence^38^ with a ^1^H transverse field strength of 100 kHz, corresponding to an effective field strength of 122 kHz. To vary the range of detection between the proton and carbon sites, ^1^H magnetization was transferred to ^13^C using a Hartmann–Hahn CP block with a variable length, with 0.1 ms for primarily one-bond correlations, 0.5 ms for intermediate range correlations, and 1.0 ms for long-range correlations.

2D ^13^C–^13^C correlation experiments were carried out using the refocused INADEQUATE scheme.^39^ The experiment was dipolar-based, using the broadband dipolar recoupling SPC5 sequence.^40^ The MAS frequencies were 10.5 kHz for the HF-treated soil sample 1 and inland plants and changed to 8 kHz for the plant samples collected at the island edge. For the direct dimension (ω_2_), the acquisition time was 17 ms for all soil and plant samples. The acquisition time of the indirect dimension (ω_1_) was 2.7 and 1.7 ms for soil and plants, respectively. The indirect dimensions of the spectra were set to 200 ppm (50–250 ppm) to effectively cover the double-quantum chemical shifts of carbohydrate and aromatic polymers. For each sample, 100 increments were collected for the indirect dimension. 320 scans were collected for the soil sample in 16 h, and 160 scans were collected for each of the two plant samples, with an experimental time of 13 and 23 h for the plants on the edge and inland, respectively. To rapidly identify the key carbohydrate components in soil, a probability map was built by extracting 412 data sets of plant carbohydrates from the Complex Carbohydrate Magnetic Resonance Database^41^ following a recently reported protocol.^42^ All ^13^C and ^1^H chemical shifts of identified polymers are documented in Table S5.

### ^14^C Dating

Prior to ^14^C dating, the soil sample was pretreated with an acid/alkali/acid solution to avoid the potential effect of the secondary carbon components (roots and bacteria) on the determined age of the sample.^26,27^ The decayed plants in the soil were used for ^14^C dating, which was calibrated to radiocarbon age (years before present, yBP) and calendar years (cal AD). The analysis was performed using the BetaCal 3.21, the INTCAL13 database, and the high probability density range method. The data set is summarized in Table S6.

### Bulk Density and Loss on Ignition

The bulk density (BD) was determined by drying the soil at 60 °C for 24 h in a muffle furnace and then calculated as oven-dry weight per unit volume at field moisture capacity.^9^ To determine the loss on ignition (LOI), the dried material was ground with a mortar and pestle and placed into a muffle furnace at 550 °C for 4 h. The mass difference before and after the combustion was divided by the original dry mass to get the percentage value of LOI ratio,^14^ which represents the relative fraction of organic matter in the sample. The results of bulk property measurements are detailed in Supporting Information.

### Total Carbon Percentage

The dried sample was ground using a mortar and pestle and sieved with a 125 μ m sieve to ensure equal particle size. 10 mg of soil was weighed into ceramic crucibles, which was placed into a total organic carbon analyzer (Shimadzu TOC SSM-5000A) to analyze the content of total carbon (TC). Information on the physiochemical properties is documented in Table S6.

## Results

### Enriched Molecular Characterization of Carbohydrates and Aromatics in Wetland Soil

**Figure 1:**
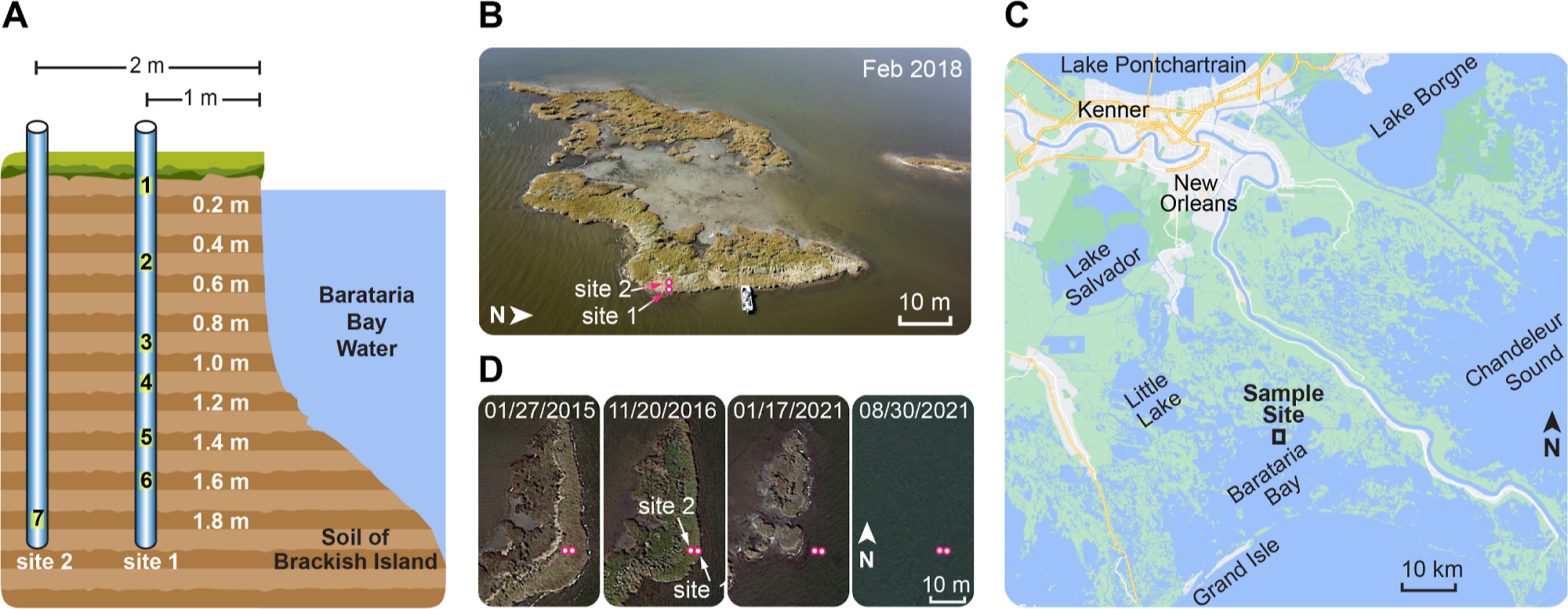
Wetland soil from a recently vanished brackish island. (A) Location and depth of seven samples used for characterization. Two poles were used to extract the soil materials, which were divided into 10 cm sections. (B) Picture of the *S. alterniflora*-dominated island with the two sample sites marked. (C) The island is 55 km southeast of New Orleans, Louisiana, USA. (D) Landscape change of the island. Positions of the two sample sites are marked using magenta circles to guide the comparison.

**Figure 2:**
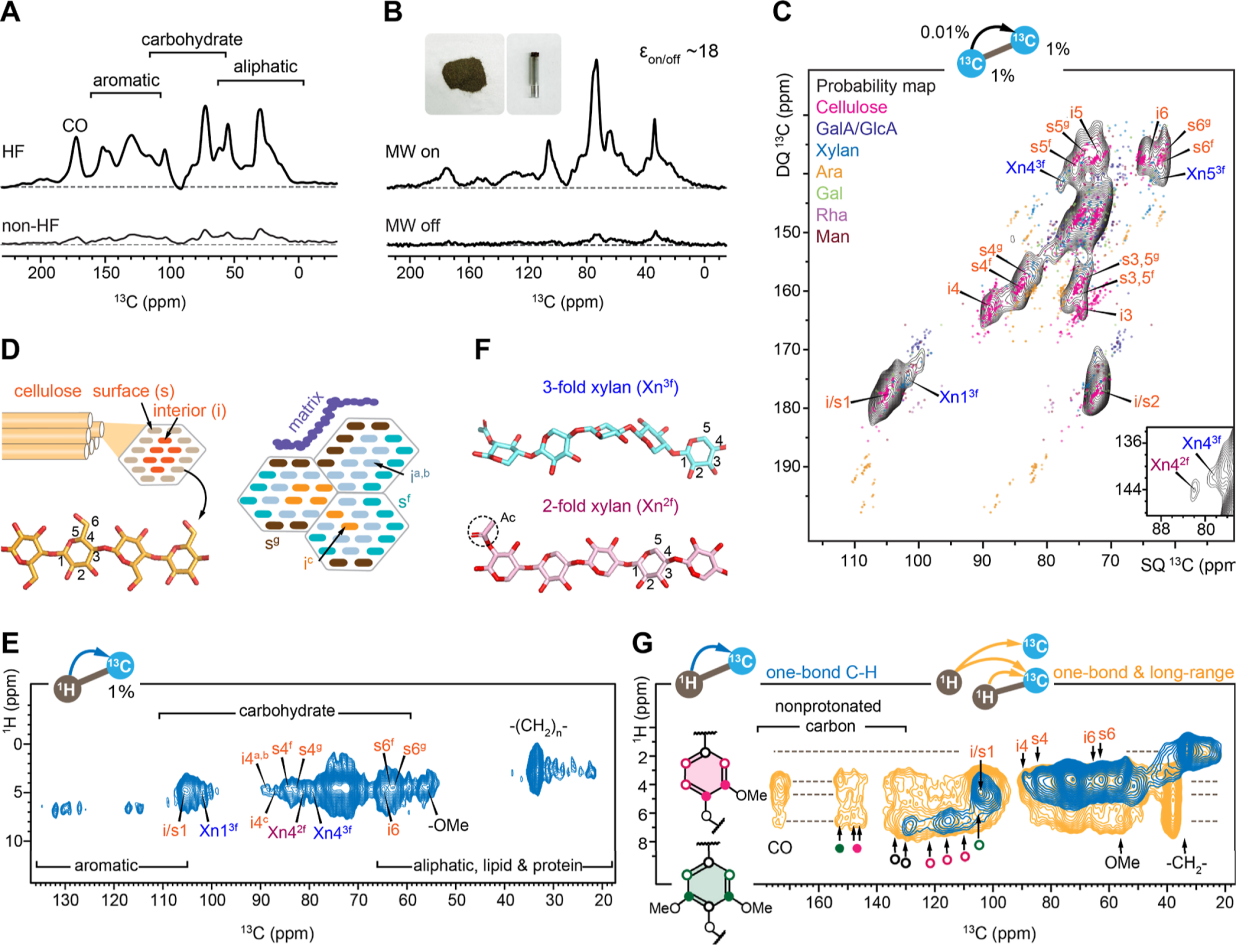
Molecular structure and spatial organization of biopolymers in wetland soil. (A) Comparison of the ^13^C multiCP spectra of the HF and non-HF treated soil sample 1 (surface layer) at room temperature. HF treatment concentrates the organic phase and gives a 5-fold sensitivity boost. (B) Comparison of the 1D ^13^C spectra with and without microwave (MW) irradiation. The sensitivity enhancement (ε_on/off_) provided by DNP is 18-fold. The inset shows pictures of the soil sample and a sapphire rotor containing the soil sample. (C) DNP-enabled 2D ^13^C–^13^C correlation spectrum (refocused J-INADEQUATE) of soil sample 1 measured in 16 h, resolving the carbon connectivity of carbohydrate components. Overlay of the measured spectrum (black) with the probability map constructed using 412 carbohydrate units from the Complex Carbohydrate Magnetic Resonance Database^41^ indicates the best match with cellulose (magenta dots). Assignments of the interior (i) and surface (s) glucan chains of cellulose microfibrils and the xylose units of 2- and 3-fold xylans (Xn^2f^ and Xn^3f^) are labeled. The inset shows the xylose carbon 4 region processed with large line-broadening to show xylan signals. (D) Representative cellulose structure with each microfibril containing 18 β-1,4-glucan chains on the surface and interior domains. Multiple microfibrils aggregate to form larger bundles that accommodate different forms of glucan chains (i^a,b^, i^c^, s^f^, and s^g^), which are further wrapped by matrix noncellulosic polymers. (E) The single-hour DNP 2D ^1^H–^13^C correlation spectrum resolves the signals from aliphatic carbons, carbohydrates, and aromatics. Key signals of polymethylene (−CH_2_−)_*n*_, methoxyl (−OCH_3_), cellulose, and xylan are labeled. (F) Structure of 2- and 3-fold xylan conformers. (G) Overlay of 2D ^1^H–^13^C correlation spectra measured with short (0.1 ms; blue) and long (1.0 ms; yellow) CP contact times. Symbolic representations correspond to the carbons in monolignol units. Dashed lines show the key ^1^H positions.

We examined seven soil samples down to almost 2 m depth (Figure 1A) collected from representative *S. alterniflora*-dominated saltmarsh (Figure 1B) in the Barataria Basin, Louisiana, located along the Gulf of Mexico coastline (Figures 1C and S1).^43,44^ Each year, Louisiana experiences a loss of over 65 km^2^ of coastal wetlands, leading to an annual release of over 1 million metric tons of stored carbon from Barataria Basin alone.^28,29^ This island underwent shoreline erosion rates of over 1.5 m y^–1^ and by 2021 had disappeared due to erosion (Figure 1D). To characterize this SOM, a two-step protocol was employed to improve the NMR sensitivity by a factor of 90, reducing experimental duration 8,100 times, essentially reducing a 22 year-long experiment down to 1 day. This was achieved by employing hydrofluoric acid (HF) treatment, a standard protocol that depleted the mineral component, concentrated SOM, and enhanced their signals by a factor of 5 (Figure 2A) without chemically perturbing its structure as described in Supporting Information,^30,45,46^ and DNP, which relies on microwave irradiation to transfer electron polarization to NMR-active nuclei in the soil,^47−51^ resulting in an additional 18 times enhancement (Figure 2B). Together, these methodological enhancements allowed for carbon connectivity to be directly mapped by a 2D ^13^C–^13^C correlation spectrum on unlabeled soil (Figure 2C), a task previously impossible by conventional techniques but now achievable over 16 h of NMR time.

The carbohydrate signals of the top 10 cm layer of soil (sample 1) were predominantly from cellulose (Figure 2C), including the glucan chains residing on both the surface and internal domains of the microfibrils (Figure 2D). Multiple forms of glucan chains are identifiable within cellulose. First, we observed two distinct sets of signals from surface chains (s^f^ and s^g^ in Figure 2C), which have been proposed to contribute to distinctly hydrated exteriors of the microfibrils, namely, the concept of hydrophobic and hydrophilic surfaces.^52^ Second, in addition to the dominant interior conformers (i^a^ and i^b^), a third form of i^c^ was also identified in a 2D ^1^H–^13^C correlation spectrum, collected within an hour (Figure 2E), with a unique C4 chemical shift of 87.5 ppm. Type-c glucan has been found in the native cellulose across many different grass and woody plant species and has been attributed to the deeply embedded and inaccessible core of the larger bundle formed by multiple microfibrils (Figure 2D).^53−55^ The identification of these five glucan types demonstrates that the original native cellulosic material was preserved in surface soil.

Further structural preservation is indicated by the unexpected identification of 2-fold and 3-fold xylan (Xn^2f^ and Xn^3f^) in the soil (inset of Figure 2C). The 2-fold and 3-fold refer to the helical screw conformation and indicate the number of sugar residues needed for finishing a 360° helical rotation along the chain (Figure 2F).^56^ Recent studies of the lignocellulosic plant biomass have revealed the distinct functions of these two xylan conformers, with the flat-ribbon 2-fold xylan coating the smooth surface of cellulose microfibrils^57−59^ and the zigzag 3-fold xylan preferentially packing with disordered aromatics, namely, the lignin domains in plants.^54,60^ Evidently, at least a portion of the structural core of plant lignocellulosic biomass is preserved, intact, in the anaerobic wetland soil.

Many noncarbohydrate molecules were also identified, including the aromatics and methoxy substitutions in lignin, the acyl chains (or polymethylene^61,62^) in lipid polymers, and other aliphatic motifs (Figure 2E). This characterization was achieved using a single-hour 2D experiment that relies on a short CP contact time (0.1 ms) to emphasize one-bond ^1^H–^13^C correlations. Protonated carbons in lignin exhibited signals in the ^13^C chemical shift range of 110–125 ppm (blue spectrum in Figure 2G).

The 105 ppm of ^13^C signals have dual contributions from both carbohydrates and aromatics, including carbon 1 of cellulose and 2-fold xylan, as well as carbons 2 and 6 of the S unit, thus showing one-bond correlations with both carbohydrate and aromatic protons. The symmetric arrangement of electron-donating methoxy groups at the 3 and 5 positions of the S unit causes substantial shielding of carbons 2 and 6, resulting in these distinctive ^13^C chemical shifts of 104–108 ppm that are well-documented in the literature for both lignin model compounds and plant lignin.^54,63,64^

The line widths of aromatic protons appear smaller than those of carbohydrate protons in Figure 2E. This effect is partly attributed to the low intensity of aromatic peaks, which generates fewer contour lines and thus narrower peaks, as well as to overlapping signals from various carbohydrates. The broad line widths for the carbohydrates also result from the structural heterogeneity of carbohydrate polymers, influenced by such factors as conformational distribution, hydrogen-bonding variations, and diverse substitution patterns.^52,65,66^

A range of nonprotonated carbons were also observed with a longer CP contact (1.0 ms) that extended the reach of ^1^H–^13^C correlation (yellow spectrum of Figure 2G; Figure S2). The spotted signals included carbonyl groups (CO) and monolignols, such as carbons 3 and 5 of the syringyl (S) unit at 154 ppm and carbons 3 and 4 of the guaiacyl (G) unit at 145–149 ppm.^55,67^ Their chemical nature was confirmed by their strong peaks in dipolar-dephasing spectra that removed all protonated carbon signals (Figure S3).^33,68,69^ The NMR observations of SOC unveiled a complex composition in terms of plant polysaccharides, lignin, and lipid polymers preserved in the soil.

### Domain Distribution of Polymers in Soil

It is noticeable that the aromatic carbons (^13^C chemical shifts of 100–140 ppm) not only show cross peaks with the aromatic protons at 6–7 ppm but also cross-talked with carbohydrate and aliphatic protons that resonate at 3–5 ppm, revealing the colocalization of aromatics, aliphatics, and carbohydrates on the nanoscale, consistent with previous work.^70,71^ This concept of molecular mixing is also supported by the cross peaks between carbohydrate carbon sites (^13^C chemical shifts of 70–110 ppm) and aromatic protons (^1^H chemical shifts of 6–7 ppm). The only exception was observed in polymethylene (−CH_2_−), which failed to show correlations with other carbons or protons, providing a clear indication of domain separation for lipid polymers. This finding corroborates earlier ssNMR results^61,70^ where polymethylene was found to form large aggregates to resist further degradation, which is a characteristic commonly shared by diverse soil materials in nature.

### Preserved Structural Core in Plant Material and Surface Soil

**Figure 3:**
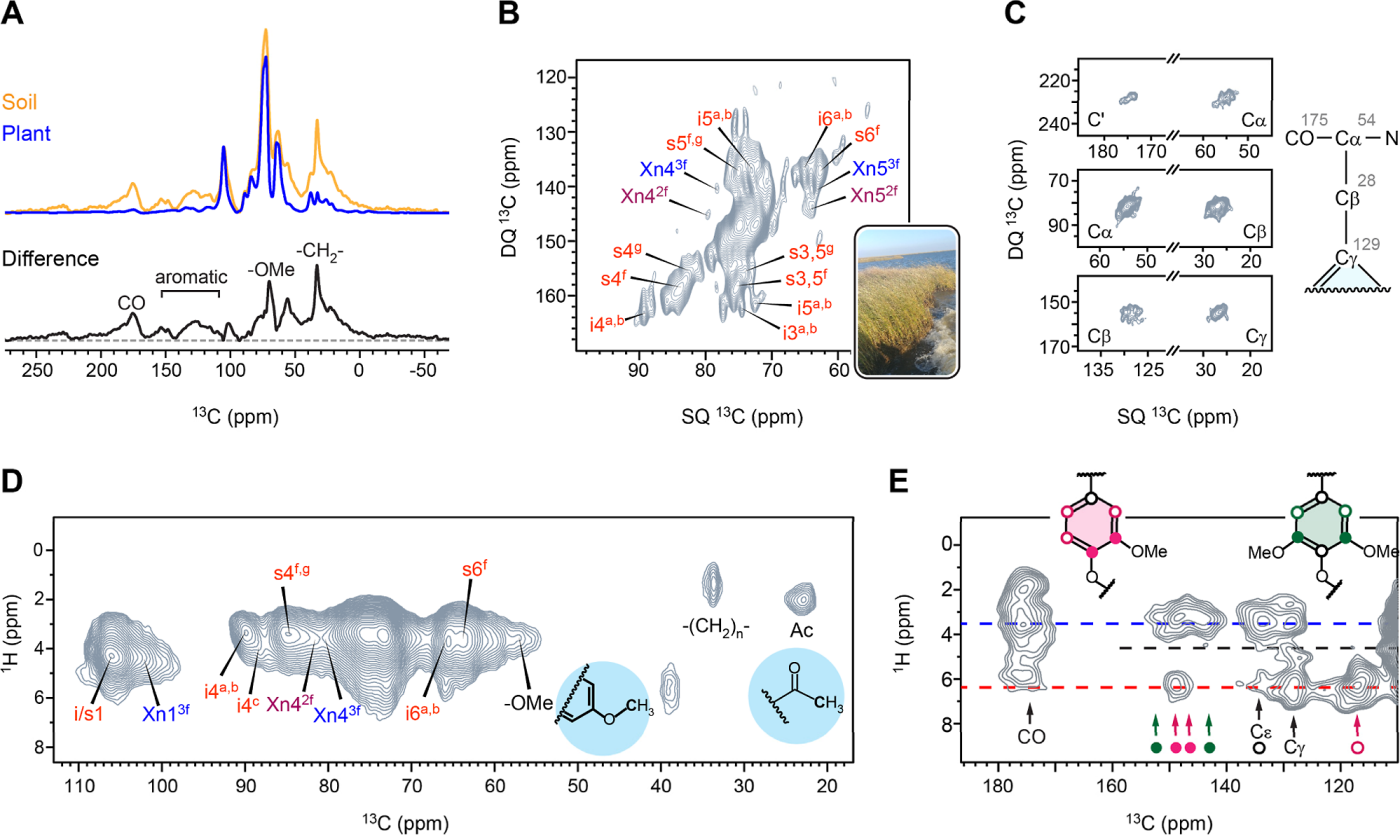
Structural features of biopolymers in plant material. (A) DNP-enhanced ^13^C spectra of surface soil (yellow; sample 1) and of the vegetation growing on the soil (blue). The bottom panel shows the difference of the two spectra, revealing a signature pattern of lignin, aliphatic, and polymethylene, as well as matrix polysaccharides. (B) The carbohydrate region of DNP-enhanced ^13^C–^13^C refocused J-INADEQUATE spectrum of the plant sample showing signals from cellulose and xylan. The inset picture shows the original plant material characterized here. (C) Aromatic amino acids resolved from the plant sample, with chemical shifts labeled on the structure. (D) 2D ^1^H–^13^C correlation spectrum of the plant sample measured using 0.1 ms CP resolving key signals of cellulose, xylan, and lipid polymers. Lignin methoxyls (−OCH_3_ or –OMe) and xylan acetyls (Ac) are also observed, with structures presented. (E) 2D ^1^H–^13^C correlation spectrum of the plant sample measured using 1 ms CP resolving signals of aromatic and carbonyl carbons from proteins and lignin. Dashed lines in blue and red annotate the key positions of methoxyl and aromatic protons, respectively. The black dashed line represents the anticipated correlations between lignin aromatic carbons and carbohydrate anomeric protons, which are notably less numerous than the signals observed in the soil, see Figure 2G.

The composition of plant detritus inputs to the soil as well as the redox status of the soil is among the key external factors that affect the rate of carbon sequestration.^72^ The plant tissues gathered at the soil collection site retain highly similar carbohydrate signals compared to those of the surface soil (Figure 3A). Cellulose crystallinity is unchanged, as evidenced by both soil and plant samples showing comparable intensity ratios between the interior cellulose C4 at 89 ppm and the surface cellulose C4 at 84 ppm. With a 24-fold DNP enhancement (Figure S4), we unambiguously detected the varied signals from multiple cellulose forms and xylan conformers in the 2D ^13^C–^13^C correlation spectrum of these plants (Figure 3B); it demonstrated a pattern similar to that of the soil spectrum. The soil exhibits elevated levels of carbonyls, methoxyls, aromatics, and aliphatics, as revealed by the difference in two parental spectra (Figure 3A). These components might have accumulated due to a slower decomposition rate when compared to carbohydrates.

Strong signals of aromatic amino acid residues have been identified that align with the chemical shifts of histidine or tryptophan (Figure 3C). These molecules are uniquely abundant in the plant material and are not present in the soil underneath it, likely due to vulnerability to rapid microbial degradation. Though the carbohydrate signals are highly consistent with the soil, the aliphatic region shows a dramatically simplified pattern (Figure 3D), with only 3 peaks from the methoxyl group of lignin, the CH_2_ groups likely from the acyl chain of lipids in the membrane or from the cutin or suberin, and the acetyl group that serves as an important modification of matrix polysaccharides, such as xylan. The aromatic region is simpler (Figure 3E). The key carbon sites of both S and G units are still detectable, with these carbons mainly correlating with the methoxyl and aromatic protons and lacking the cross peak with the anomeric protons of carbohydrates at 4.3–4.5 ppm, suggesting substantially reduced interactions between carbohydrates and aromatic polymers.

Despite the conserved structures of individual carbohydrate and lignin components, the SOC has two unique structural features that are absent in the original plant materials. First, noncarbohydrate components are highly concentrated, which is likely caused by the faster degradation rate of polysaccharides compared with lignin and polymethylene polymers. Phenols serve as an antioxidant during degradation reactions, while polymethylene polymers contain crystalline domains of aliphatic chains, conferring these polymers with high stability.^73,74^ This trend was also confirmed by the analysis of another plant sample collected 30 m inland on the same island (Figure S5). Second, the aromatics and carbohydrates are more tightly packed in the surface soil than in plants. This finding might originate from the faster decay of primary cell walls that contain only cellulose and soft matrix polysaccharides but do not contain lignin and/or the removal of intra- and extracellular components leading to tighter packing of residual lignocellulosic components.

### Mapping Carbon Composition and Packing along Depth

**Figure 4:**
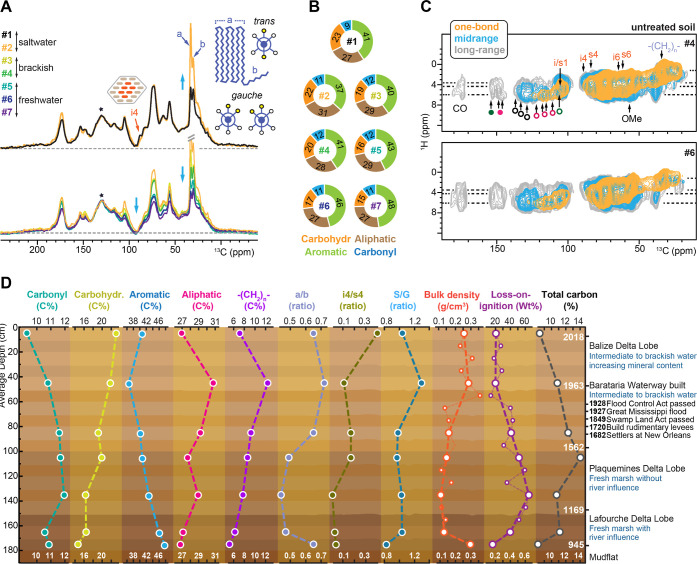
Structural changes of SOM in relation to depth. (A) Overlay of 1D semiquantitative ^13^C spectra of seven soil samples with normalization by the major aromatic peak (asterisk). The asterisk denotes the primary aromatic peak, which arises from overlapping signals of S and G, and possibly from other lignin units, such as *p*-hydroxyphenyl and ferulate. Representative signals underlying this primary peak include, but are not limited to, S1, G1/6, H1/2/6, and FA1/6. The top panel compares samples 1 and 2, and the bottom panel includes samples 2–7. The two key peaks (types a and b) of polymethylenes are marked, with an illustration of the crystalline all-*trans* domain and the amorphous domain that include both trans and gauche conformations. Three conformers, gauche(+), gauche(−), and *trans*, are illustrated, with methylene groups represented as yellow circles and hydrogen atoms as open circles. The wetland condition of each soil sample (saltwater, brackish, or freshwater) is also labeled. (B) Estimation of the fraction of four major carbon types. The details of spectral deconvolution are documented in Figure S6 and are calibrated by comparison with quantitative DP spectra documented in Figure S7. (C) ^1^H–^13^C correlation spectra of two untreated soil samples measured with 0.1 ms (yellow), 0.5 ms (light blue), and 1 ms (gray) CP contact times. (D) Molecular and physical evolution of wetland soil over time. The figure depicts a geological timeline through soil depth based on ^14^C dating results and historic events in the area. Molecular profiling of 7 soil samples reveals the content of major carbon types. Detailed information differentiates different types of polymethylene carbons, cellulose chains, and monolignol units. BD and LOI measurements were also taken on 18 soil samples immediately following collection. A higher BD indicates a higher mineral content, while a lower LOI % suggests a lower organic matter content.

The molecular composition of the soil matter changes with the depth. In the semiquantitative 1D ^13^C spectra,^31^ the content of polymethylene carbons, marked by the intensities of two adjacent peaks at 33 and 31 ppm, increased substantially from sample 1 (0–10 cm interval) to sample 2 (40–50 cm interval), as shown in Figure 4A. These two peaks in polymethylene, also observed in many other SOM samples, correspond to crystalline (CH_2_)_*n*_ chains in all-*trans* conformation (type a; 33 ppm) and amorphous regions accommodating both *trans* and gauche conformations without long-range order (type b, 31 ppm), as demonstrated by Schmidt-Rohr and colleagues.^61^ The self-aggregated nature and limited accessibility of polymethylene might have prevented it from being degraded after deposition in the soil. However, when normalized to the aromatic signal, polymethylene together with carbohydrates and carbonyls decreases sequentially as one moves deeper in the soil profile (samples 2–7; age range from 1963 to 945 AD). These spectral observations were further confirmed through a deconvolution protocol applied to 1D ^13^C multiCP spectra (Figure S6), using the carbon sites resolved from a high-resolution 2D data set.

Despite the use of multiCP experiment, there was still a preferential detection of carbohydrate and aliphatic carbons over aromatic and carbonyl groups; therefore, a quantitative ^13^C DP experiment with long recycle delays of 90 s was conducted to obtain calibration factors for carbohydrate and aliphatic carbons (Figure S7).^75,76^ After intensity calibration, carbohydrates exhibit a substantial reduction in their proportion, decreasing from 23% in sample 1 to 15% in sample 7, while the content of aromatics is enriched from 41% to 48% when moving deeper from the surface (Figure 4B). It is notable that the carbohydrates have survived degradation after 1000 years despite the fact that the soil has substantial extracellular enzyme activity, highlighting the protection of these easily degraded compounds by tight packing with phenolic polymers.^27^ While this approach provides an overview of the molecules present in the sample, more quantitative techniques—such as carbon counting via intensity calibration with model compounds^75,76^—could enable a more accurate analysis of the polymer composition.

Regarding the ratio between carbohydrate and aromatic moieties, there is a vertical distribution based on salinity as the wetland converted from freshwater, to brackish, to a current-day saltmarsh environment (Figure 4A).^77^ A similar trend was observed regarding the polymethylene-to-aromatics ratio moving from deeper soil to the present-day surface. This finding suggests that as the sea level rises, salinity and resulting shifts in plant species are likely to affect how the sequestered carbon is stored and ultimately degraded in the soil.

Native, untreated soil samples were also investigated by using DNP-enhanced 2D ^1^H–^13^C correlation experiments (Figure 4C). These nontreated soil samples received a DNP enhancement of 10–30-fold (Figure S8). It is intriguing that the spectral pattern was consistently maintained in the untreated samples 4 and 6 (Figure 4C) and in both HF-treated and untreated materials of sample 1 (Figures 2G and S9); therefore, the HF treatment did not perturb the native structure of the SOC core. This similarity is also an indication that at least a significant portion of the lignocellulosic cores were preserved, supported by the observation of internal cellulose, whose carbon 4 (i4) shows a major decline starting from sample 2 (Figure 4A) but still exhibits some weak signals in samples 4 and 6 (Figure 4C). While most cellulose was decomposed rapidly in the surface layers, a fraction of these crystalline cores were still preserved for centuries in this wetland soil.

The presence of plant carbohydrates within these wetland soil samples was confirmed by successfully isolating plant-derived materials directly from soil samples at depths of 120–130 cm, 150–160 cm, and 170–180 cm (Figure S10). Moreover, the observed 89 ppm ^13^C signals (Figure S9) are characteristic of interior chains or crystalline regions within cellulose microfibrils, where glucan chains are stabilized by hydrogen bonding within the microfibril structure. These chains adopt a trans–gauche conformation of the hydroxymethyl group,^52^ a conformation that is rarely seen in other carbohydrates. Additionally, the spectral pattern of the carbohydrate region remained largely consistent from sample 2 to sample 7, suggesting a similar structure for the carbohydrates detected here across samples despite a sequential decrease in intensity.

Two key biogeochemical conditions within coastal wetlands enable the long-term preservation of biopolymeric plant material at depth, spanning centuries to millennia. First, oxygen penetration is significantly restricted; beyond the surface layer, water saturation depletes available oxygen quickly, and due to the ∼10,000-fold slower diffusion rate of oxygen in water compared to air, it is not replenished. This creates anaerobic conditions within the soil column, unlike the conditions found in upland, well-drained soils. Second, coastal wetlands maintain a poised or relatively high buffered redox potential that is much more reduced than that of upland soils. Together, these factors significantly slow the degradation of plant biopolymers in coastal wetland soils, resulting in preservation over extended time scales.^11^

The cross peak at the ^13^C chemical shift of 38 ppm and ^1^H chemical shift of 5.4–5.8 ppm remains unassigned. This signal was initially detected in the intact plant sample (Figure 3D) and persisted in the soil sample (Figure 4C). It may originate from a plant metabolite, such as 3-buten-1-ol, or from other plant molecules or polymers that are currently unidentified. Given the molecule’s resistance to degradation and its consistent presence in both plant and soil samples, it was noted but not emphasized in the analysis.

The close spatial proximities between aromatics and carbohydrates observed in samples 4 and 6 (Figure 4C) resemble those identified in the surface soil (Figure 2G). Polymethylene also shows the same self-aggregation features in these deeper samples. Therefore, the decomposition of SOM did not happen homogeneously. Some biopolymeric structural cores, such as self-aggregated polymethylene and densely packed lignin–polysaccharide domains, have efficiently withstood microbial degradation and maintained their original structure and physical packing after approximately 500 years (at 100 cm depth) and even up to 1000 years (at 180 cm depth). This preservation has been maintained despite the presence of microbial extracellular enzymes throughout the profile.^27^

### Natural and Anthropogenic Influences on Carbon Speciation over a Millennium

The observed nondirectional changes of the molecular composition and structure (Figure 4D) are not expected based on conventional soil aging and humification or considering the activity of microbial communities at depth. As tracked by the ^14^C dating, the soil material collected across the ∼2 m depth covers a geological timeline of 11 centuries;^26,27^ therefore, the interplays of the switching delta lobes and water salinity in the Barataria Basin should also play a key role. The Mississippi River watershed is the dominant surface hydrologic feature in North America, which collects runoff from 40% of the continental US between the Rocky Mountain region and the Appalachian Mountains.^77^ River deltas are dynamic systems with continually shifting lobe formation and abandonment over time.^77^ Therefore, these environmental shifts over time can influence the physical and chemical characteristics of the accreted carbon pool based on hydrodynamics and salinity. One anthropogenic driver has been the construction of a system of continuous river levees in the lower river over the past century, essentially separating the coastal basins from the river, preventing the historical freshwater and sediment subsidies from occurring.^78^

The continuing decrease of LOI and TC and the gradual increase of BD from 1 m depth to the surface of the soil are due to marsh fragmentation (Figure 4D; see Supporting Information). As the continuous marsh platform begins to erode from all edges, the interior of the marsh is located closer to the shoreline. Consequently, fine-grained sediments present in the bay are transported into the marsh during storm and tidal events.^79,80^ The soil at 40 cm depth formed under saline conditions also shows the most unique chemical characteristics that violate the trends on the molecular level. It shows the highest content of aliphatics and polymethylene, and polymethylene has a unique structure that is rich in the carbon site resonating at 34 ppm (named type-a polymethylene; the crystalline domain in all-*trans* conformation).^61^ Lignin contains a high level of S monolignols with a high degree of methoxy substitutions. Cellulose crystallinity also becomes low: only approximately one-tenth of glucan chains are now situated in a crystalline interior environment, while the remaining majority are disordered.

## Discussion

It is imperative to understand the connection between the chemical stability of wetland SOC and its carbon structure, given wetlands’ crucial role in global carbon stocks and their ability to sequester more carbon per unit area compared to other soil types.^1,6^ It is also crucial to differentiate between molecular and carbon speciation, microbial transformation, and preservation^18,19^ when considering SOC persistence as the sea level continues to rise globally.^8,13^ In this pursuit, ^13^C ssNMR spectroscopy,^81−83^ along with the sensitivity enhancement by DNP,^36,47^ has been introduced to minimize the biases introduced by the extraction, solubilization, and relaxation present in solution NMR (Supporting Information), while leveraging the molecular view NMR spectroscopy allows, especially multidimensional techniques. This has allowed new insights into SOC sequestration and the importance of preservation for organic-rich and POM-dominated blue carbon systems.

This study reveals robust preservation of the polymeric assembly in the top 10 cm of soil echoing the core structure of plant parent materials, evident in the comparable interior and surface cellulose signals, the preservation of multiple forms of cellulose, xylan conformers, and both the S and G monolignols in lignin (Figure 2C,E,F). This result can be attributed to the tight packing of some lignin and carbohydrate components, which could be induced by the decay of bulky cell wall cellulose and soft matrix polysaccharides, as evidenced by the lower content of these moieties within the whole soil (Figure 3A). Further evidence of decay taking place in parallel with preservation is the absence of aromatic amino acids within the SOM (Figure 3C). This new insight allows for a refocusing on the concept of molecular preservation in the form of the conservation of the structural core of parent biopolymers, i.e., recalcitrance, as an important component of carbon storage, especially for high organic matter soil systems, such as we find in wetlands, and counters the concept that free POM, as a whole, is a less than stable form of carbon.^84,85^

The preservation of recalcitrant lignocellulosic domains in soil POM involves maintaining both the molecular structure and the supramolecular assembly of participating biopolymers (Figure 4C). This concurs with the biomolecular transformation of more-accessible molecules on the millennium time scale. The rapid decay of carbohydrates can be explained by the preferential utilization by microbes over other molecules, such as aromatic compounds, as both an energy and a nutrient source under the anaerobic soil conditions.^84^ The better preservation of aromatics over polymethylene is likely related to the reduced soil conditions. While soil microbes can produce extracellular compounds such as phenol oxidase, these metalloenzymes require oxygen to oxidize phenol compounds. Hence, in the anaerobic wetland soil profile, these compounds are stable.^10^ It has been found that high phenolic compounds strongly inhibit hydrolases further muting microbial decomposition of SOM,^86^ thus, we posit that to a limited extent, additional aromatic moieties are synthesized by biotic/abiotic processing of the loosely associated lignocellulose.^68,87,88^

The observed changes in the tightness of POM packing offer an unprecedented understanding of its persistence, in conjunction with the “enzyme latch” preservation mechanisms.^86^ First, it allows new insights into how carbohydrate moieties are preserved as it presents a mechanism for reducing surface volume and reducing the accessibility of microbial entities to these more easily degraded carbohydrates under anaerobic wetland soils conditions. In terms of the “enzyme latch” mechanisms,^86^ its ubiquitous application has recently been challenged by the application and coupling of advanced metaproteome, metabolite, and mass spectroscopy methods,^89^ in which it has been shown that, even under anoxic conditions, (i) polyphenols are depolymerized, (ii) soil microbial function is maintained or even enhanced by the polyphenol degradation, with this degradation resulting in microbial community changes, and (iii) a cascade of degradation steps take place and are driven by different enzymes and associated microorganisms. These studies, in combination with the current results, suggest two parallel carbon molecular preservation wetland pathways: one through the preservation of core structures and the other through decomposition and repolymerization. This convergence of in-depth analyses of a limited sample set, like the current one and recent research,^89^ offers a more nuanced understanding of preservation during carbon sequestration, which would not have been possible by bulk analysis of large sample sets.

The DNP techniques employed here have also made it possible to obtain an in-depth view of the chemical and physical structures of biomacromolecules in many other coastal wetlands and organic-rich soil systems, with caution that these results are to be viewed qualitatively. Coupled with extensive bulk characterization data, this approach holds promise for advancing SOC analysis. Given the significant impact of plant traits on belowground biomass and marsh resilience to sea-level rise,^90^ such new analytical capabilities can also inspire future studies to bridge the gap between climate-induced plant species migration with the resulting SOC stability and wetland resilience to erosion.

The dynamics of deltaic systems on C sequestration were revealed through trends within the ^13^C multiCP data, with major transformations found to occur within the SOC pool over a millennium (Figure 4A,D). The first important takeaway is that the conditions of the wetland soil under which the SOC is initially preserved play a major role in the decomposition of the SOC pool. This is evidenced by an increased preservation of carbohydrates when the wetland transitioned from freshwater to brackish water and to a saltwater-dominated wetland in conjunction with aging (Figure 4A); the same general trend can also be seen regarding polymethylene. The relative proportion of preservation only changed with time with the change in the depositional environment, highlighting the environmental controls on plant species as the dominant factor.

The initial transition from a freshwater to brackish water wetland was due to geological influences in the form of lobe transition (Lafourche to Plaquemine lobe and Plaquemine to Balize lobe, respectively), while the transition from brackish water to a saltmarsh was influenced by both a geological lobe relocation and levee construction, starving the wetlands of freshwater and sediment inputs.^77^

On aggregate, SOC sequestration in the studied coastal wetland can be viewed as a combination of molecular—including biomolecular—preservation, recalcitrant carbon, and carbon stabilization through dynamic carbon speciation (decomposition and repolymerization). The hydrogeomorphic setting changed over the 1000 years during which this sequestration has taken place, switched from an active freshwater delta to an abandoned freshwater delta lobe and then to a brackish and, eventually, a saltmarsh system, as the sea level has continued to rise and the river moved away. Despite these drastic surface changes, preservation via tighter packing of parent biopolymers has been consistent over time. A new framework of terminology can be derived, in which preservation can be viewed as molecular preservation and sequestration can be viewed as carbon storage regardless of speciation, with preservation being a subcategory of sequestration. This study provides strong evidence for giving significant weight to POM, just as what has been done for MAOM in regard to global SOC management,^20^ with POM being a major focus for organic soils, such as wetlands which contain ∼1/3 of the planet’s SOC. Recalcitrance should also be a major part of the focus within the preservation of high organic soil, especially as this preserved SOC becomes quickly processed and converted to carbon dioxide when exposed to highly oxygenated saline water, hence precluding methane formation, due to erosion.^13,14,91^ Therefore, POM and molecular recalcitrance, including biopolymeric structures, via structural tightening in an oxygen-poor environment, as well as carbon speciation via decomposition and repolymerization (humification), are among the important drivers in SOC sequestration for about one-third or more of the planet’s SOC pool, with implication for greenhouse gas emissions when these banked (centuries to millennia) but vulnerable carbon pools are introduced to oxygen-rich waters due to erosion such as induced by sea-level rise. The loss of such carbon globally could overwhelm any reduction in carbon emissions humans may enact and severely impact the atmospheric carbon pool and its modeling.

## Supporting Information Available

HF treatment, DNP, bulk properties, SOM, granularity, and soil characterization techniques; figures and tables related to sample preparation; additional DNP, NMR, and EPR data; and spectral analysis (PDF)

## Abbreviations

CP: cross-polarization
BD: bulk density
DNP: dynamic nuclear polarization
LOI: loss on ignition
TC: total carbon
MAOM: mineral-associated organic matter
MAS: magic-angle spinning
NQS: nonquaternary suppression
SOC: soil organic matter
POM: particulate organic matter
