# Supplementary material for: Enriched Molecular-Level View of Saline Wetland Soil Carbon by Sensitivity-Enhanced Solid-State NMR
Source: J Am Chem Soc. 2024 Dec 19;147(1):519–31. doi: 10.1021/jacs.4c11830 (PMC11726556; doi:10.1021/jacs.4c11830)
Supplement: Supplementary file 1 — ja4c11830_si_001.pdf [file ja4c11830_si_001.pdf]

Supplementary Information

# An Enriched Molecular-Level View of Saline Wetland Soil Carbon by Sensitivity-Enhanced Solid-State NMR

Wancheng Zhao[1†‡], Elizabeth C Thomas[2‡], Debkumar Debnath[1], Faith J. Scott[3], Frederic Mentink-Vigier[3], John R. White[4, 5], Robert L. Cook[2*], Tuo Wang[1*]

[1] Department of Chemistry, Michigan State University, East Lansing, MI 48824, USA
[2] Department of Chemistry, Louisiana State University, Baton Rouge, LA 70803, USA
[3] National High Magnetic Field Laboratory, Florida State University, Tallahassee, FL 23310, USA
[4] Department of Oceanography & Coastal Sciences, Louisiana State University, Baton Rouge, LA 70803, USA
[5] Coastal Studies Institute, Louisiana State University, Baton Rouge, LA 70803, USA

[‡] These authors contributed equally
[†] Present address: Laboratory of Chemical Physics, National Institute of Diabetes and Digestive and Kidney Diseases, National Institutes of Health, Bethesda, MD 20892, USA

*Corresponding authors. Email: wangtuo1@msu.edu; rlcook@lsu.edu

**This PDF file includes:**

Supplementary Text

Figs. S1 to S10

Tables S1 to S6

Supplementary References

## Supplementary Text

Effect of HF treatment on soil

The application of 2% HF solution efficiently depleted iron and mineral component in soil, allowing for a substantially higher concentration of SOM within a MAS rotor. Comparative analysis of ssNMR spectra showed no noticeable difference between HF-treated and untreated samples, whether at room temperature (**Fig. 1A**) or at the cryogenic temperature used for DNP measurements (**fig. S8A**). Discrepancies were observed between MultiCP and DNP-enhanced CP spectra (**Fig. 1A, B**), which were not caused by HF treatment but resulted from the application of higher magnetic fields and MAS frequencies, reducing the signals of aromatic motifs with significant chemical shift anisotropy (CSA).

Inhomogeneity of DNP in soil samples

For DNP measurements, soil materials were doped with 10 mM of a stable biradical named AsymPolPok[1]. This biradical is water-soluble and has a small dimension of 1-2 nm, enabling penetration in porous materials. When the electron polarization is transferred to $^{1}H$ in the DNP matrix, it relies on $^{1}H$-$^{1}H$ relayed transfer, ensuring efficient and homogenous polarization across a range of at least a hundred nanometers, as recently reported[2]. Typically, it is straightforward to achieve homogeneous polarization throughout the sample. However, within each soil sample, polarization levels varied among molecules and structural motifs. In the case of HF-treated sample 1, all carbohydrate signals exhibited a uniform 18-fold enhancement, whereas aromatic and aliphatic carbons experienced a comparatively lower enhancement of 7-9-fold (**fig. S8B**). Hence, the predominant portions of carbohydrates appear to be more accessible, which may partially explain their accelerated degradation rate through microbial processes in soil. Alternatively, carbohydrates may exhibit enhanced retention of solvent and biradicals, probably due to the rich polar functionalities present in carbohydrates. Similar patterns were observed in non-treated soil sample 1 (**fig. S8C**) and non-treated samples #4 and #6 (**fig. S8E, F**), where carbohydrates consistently exhibited the highest enhancement, while other components displayed slightly lower enhancements. Remarkably, the most effective DNP performance was noted in non-treated soil sample 4, demonstrating a 33-fold enhancement for carbohydrates and a 23-fold enhancement for all other molecules (**fig. S8E**). Additionally, it is noteworthy that even in plant samples, polymethylene peaks exhibited minimal DNP enhancement (**fig. S4B, D**), indicating the presence of lipid polymers in self-aggregated domains that are challenging to penetrate, not only in soil but also in plants. This could also be due to faster $^{1}H$ relaxation, leading to inadequate preservation of hyperpolarization.

Bulk properties of wetland soil samples

During the Lafourche delta lobe formation, the Mississippi River ran through Barataria Bay and deposited silt and mineral matter into the surrounding wetland soils. The soil at 1.5-1.8 m depth formed during that period, exhibiting very low loss-on-ignition (LOI) ratios of 1.16-1.40 and total carbon (TC) percentage of 8.1-11.2%, but a high bulk density (BD) of 0.14-0.31 g/cm$^{3}$ (**Table S6**). High BD indicates a high concentrations of large mineral particles, with the soil expected to erode more slowly[3], while a low LOI ratio represent a low content of organic matter. When it came to the Plaquemines delta lobe, the Barataria Bay became a fresh marsh and expected to contain more organic matter[4-6]. Consistently, the soil at the depth of 1-1.5 m showed a high LOI ratio of 1.51-1.65, a high TC percentage of 10.7-14.0%, and a low BD of 0.11-0.18 g/cm$^{3}$. The geological and historical timestamps explain the observed changes in the chemical composition of soil organic

matter. Across all geological stages listed here, the soil formed during the Lafourche delta lobe is featured with a high content of aromatic carbons and a low amount of carbohydrates. This trend has been flipped in the soil associated with the Balize delta lobe.

An incomplete molecular characterization of SOM

Advances in analytical instrumentation, in terms of resolution, sensitivity, and less harsh sample pre-treatment, in concert with some highly insightful experimental design, has allowed for new data and insights to emerge and a rethinking of SOM preservation. While there is no argument that these new techniques have allowed a major leap forward in our understanding of SOM and the associated SOC fraction, care must be taken when extrapolating the data interpretation to SOM as a whole. In addition to the bias on upland/mineral soil, as described in the Introduction, two guiding principles are also useful: 1) granularity (speciation versus molecular) and 2) bias on extraction and small molecule and the need for complementary characterization techniques.

Granularity

Methods that interrogate whole soils, to date, provide data at an elemental to moiety level, e.g., aliphatic (alkyl), carbohydrate (O-alkyl), aromatic (aryl), aromatic with oxygen functional groups (O-aryl), phenolic, carbonyl group, *etc*., but not at the molecular level, which identifies exact molecular structures. This challenge is a result of the complexity of SOM as well as the limited resolution of each individual technique applied to whole soils to date. Some of the key techniques include Nano Secondary Ion Mass Spectrometry (NanoSIMS), near-edge X-ray spectromicroscopy, and NMR, including 2D methods[7-12]. Consequently, unlike in other research fields, while we are able to interrogate a system and gain a general moiety (structural categories) level understanding, we are unable to ask direct questions regarding the more informative and scalable molecular level.

Need for complementary technique for soil characterization

Two techniques that have been able to overcome the granularity limit mentioned above are FT-ICR-MS and multidimensional liquid state NMR, each with their own limitations brought about by three biases. The *extraction bias* arises from the SOM being extracted and, since all too often, such extraction is incomplete[13], thus these techniques are blind to the non-extracted fraction of SOM. The *solubility bias* stems from the fact that not all SOM, even extracted SOM, is equally soluble, with the more soluble fraction being more highly weighted. The third bias is the *small molecule bias*. For liquid state NMR, this bias is a result of molecular rotation, which leads to differences in relaxation behavior, especially $T_2$ relaxation, with smaller molecules yielding sharper peaks and hence more intense signals compared to larger molecules that yield broader spectral peaks and tend to be lost due to the broad peak convolution for samples as complex as SOM extracts. On the FT-ICR-MS front[14,15], this small molecule bias, all other things being equal, results in a small molecule ionization preference. This means that the high-resolution FT-ICR and liquid state NMR data are biased towards smaller molecules, with an understanding that the absence of evidence of larger molecules does not mean they are not there. In this context, DNP-enabled high-resolution ssNMR can serve as a complementary technique to provide information on the large macromolecules in soil materials.

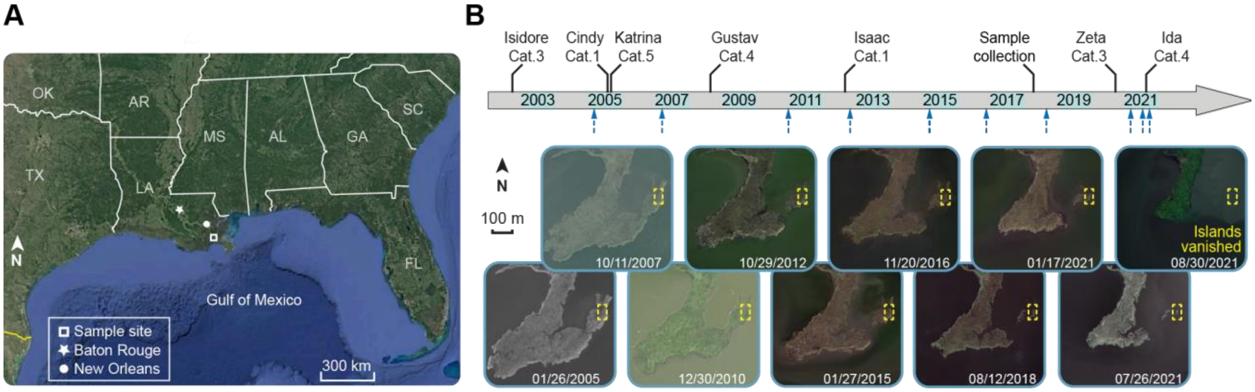


**Fig. S1. Location and landscape of the brackish island for sample collection.** (**A**) The island is 55 km southeast of New Orleans, and 160 km away from Baton Rouge, the capital city of the state of Louisiana, USA. Soil materials were collected in February 2018 from a brackish island in Barataria Bay (GPS coordinates: 29°26'36.9"N, 89°53'59.0"W). (**B**) Timetable summarizing the landscape change of the island and adjacent lands over two decades. The catastrophic hurricanes that affected this island and the category (Cat.) numbers of these hurricanes are labeled. Blue dashline arrows indicate the ten time points where pictures of the landscape are provided.

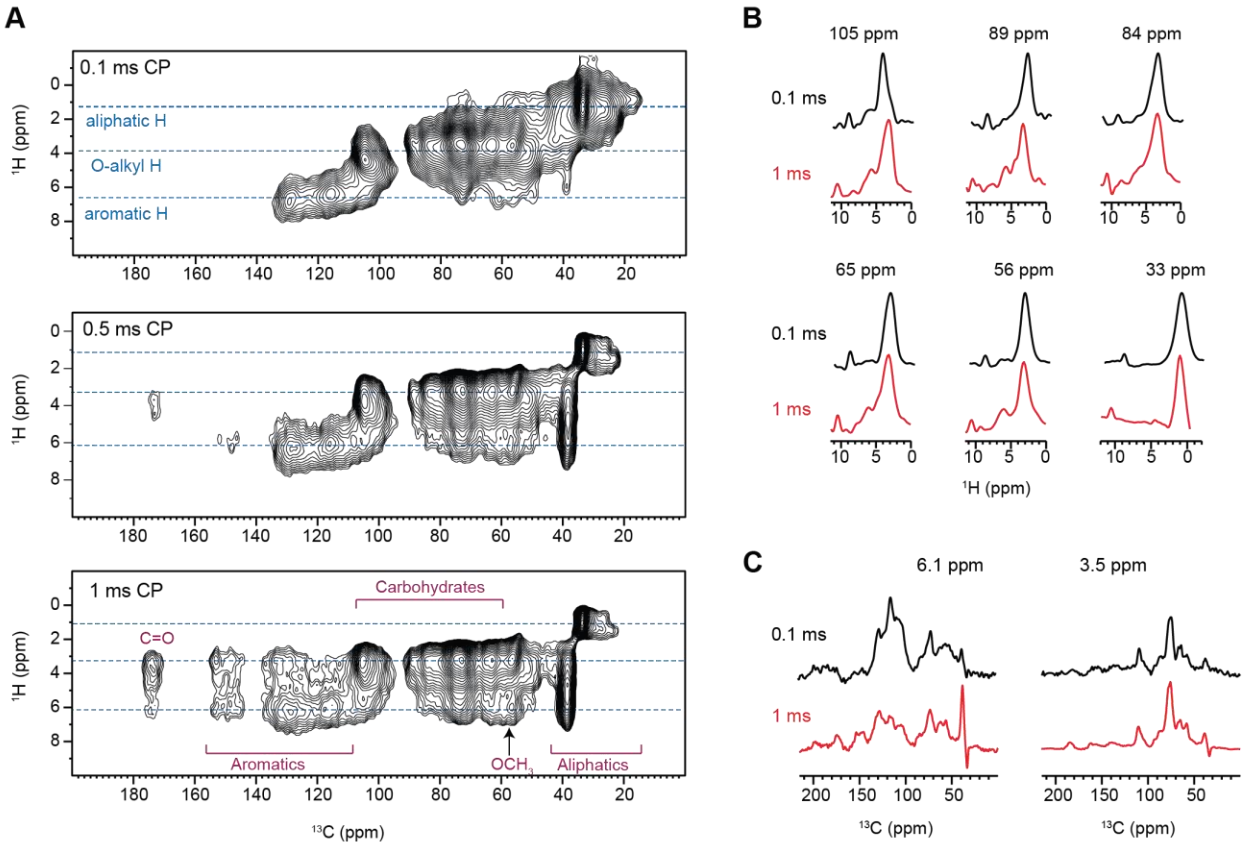


**Fig. S2. DNP 2D $^{1}$H-$^{13}$C correlation spectra of unlabeled HF treated soil.** (**A**) 2D $^{1}$H-$^{13}$C correlation spectra measured with short (0.1 ms), medium (0.5 ms), and long (1.0 ms) of CP contact times of unlabeled soil sample 1. The blue dash lines show the key proton positions of aliphatics, carbohydrates, and aromatics. The spectrum with 1 ms CP shows intermolecular cross peaks between aromatics and carbohydrates. (**B**) Representative 1H cross sections extracted at different carbon sites from the 0.1 ms (black) and 1 ms (red) CP contact times. (**C**) Representative $^{13}$C cross sections from the 0.1 ms (black) and 1 ms (red) CP contact times. The spectra were measured at 10.5 kHz.

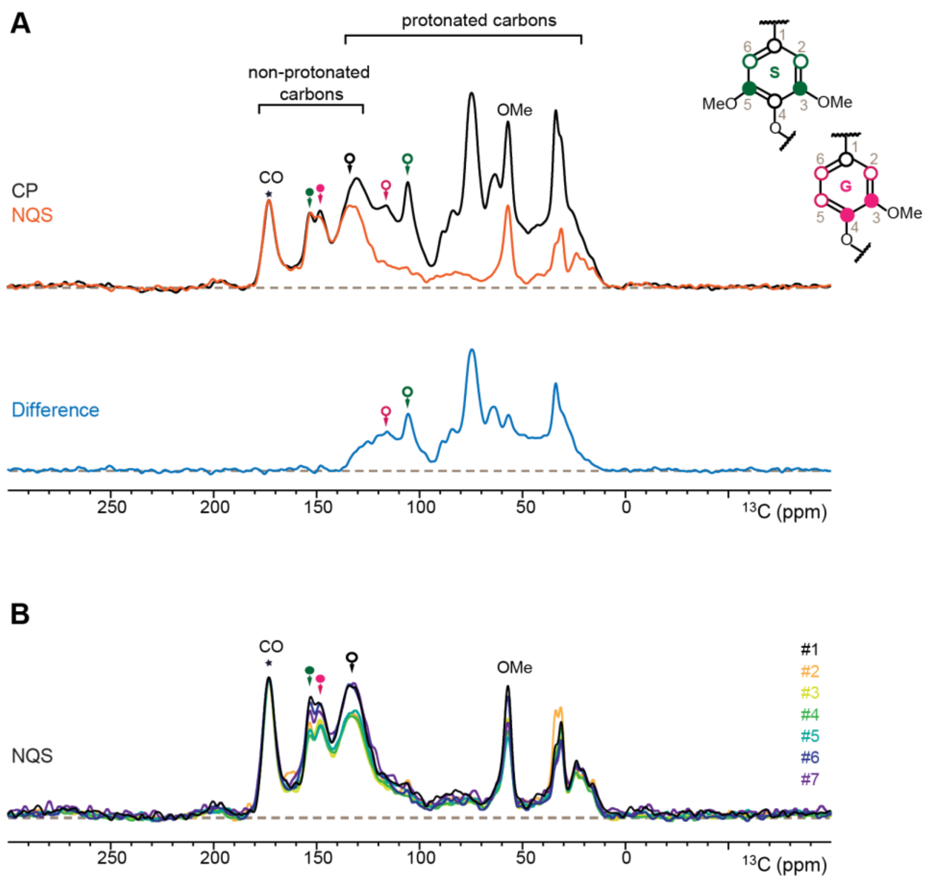


**Fig. S3. 1D non-quaternary suppression detecting non-protonated carbons.** (**A**) Comparison of $^{13}C$ CP and NQS spectra of the HF treated soil sample #1 at room temperature. The difference spectrum shows only protonated carbons. (**B**) Overlay of 1D NQS $^{13}C$ spectra of seven soil samples. All spectra are normalized by the CO peak (asterisk). QNS spectra mainly shows non-protonated carbons, with methyl carbons as an exception due to their rapid molecular motions.

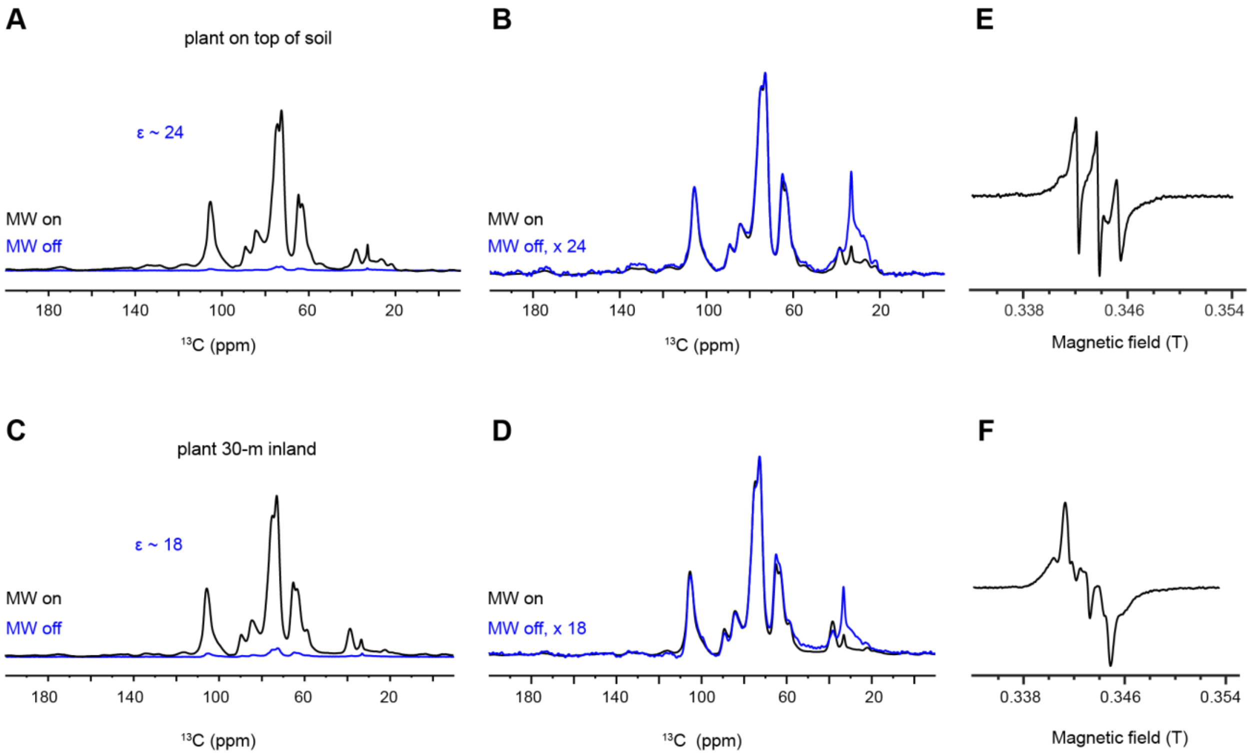


**Fig. S4. DNP and EPR spectra of plant samples.** (**A**) DNP enhances the sensitivity by 24-fold for the plant on top of soil (on the edge of the island). (**B**) Magnification of the microwave (MW) off spectra showed overall consistent pattern with the MW-on spectra, revealing homogeneous polarization by DNP, except for the polymethylene peaks. (**C**) and (**D**) The 30 m inland plant sample also showed 18-fold of DNP enhancement, with homogeneous DNP of carbohydrates and aromatics as shown in panel. (**E**) and (**F**) Room temperature EPR spectra of AsymPolPOK ($D_2O/H_2O$, 90/10 Vol%) at 9.6 GHz for these inland plants.

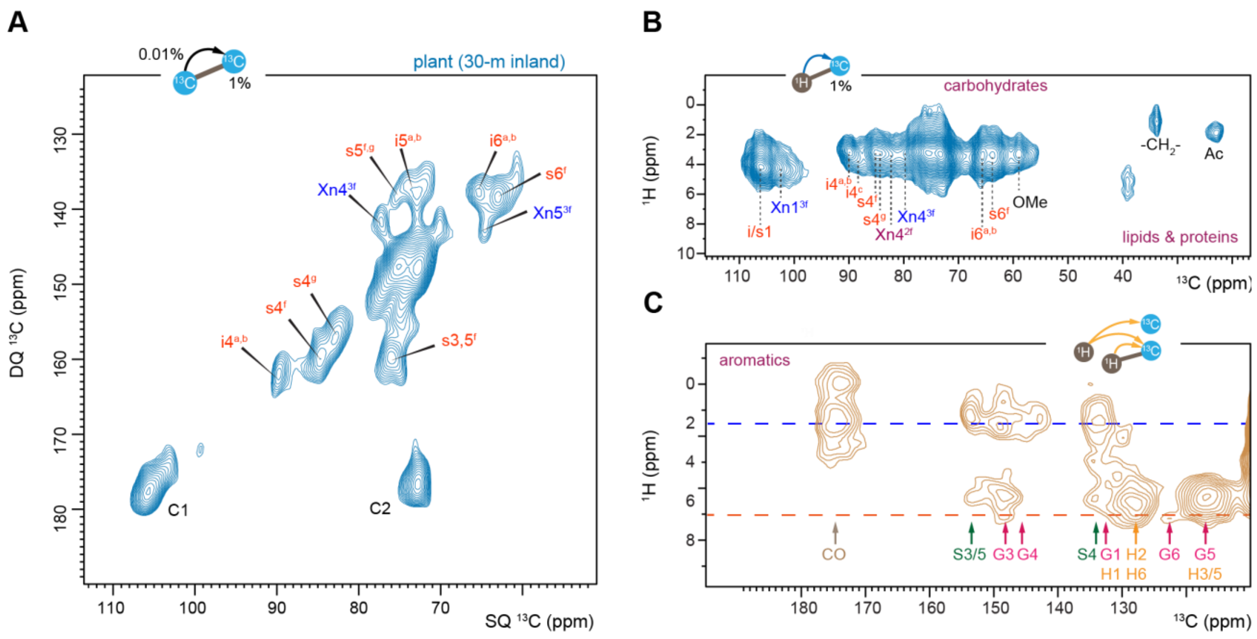


**Fig. S5. 2D $^{13}$C/$^{1}$H-$^{13}$C spectra of unlabeled plants 30-m inland.** (**A**) Carbohydrate region of DNP enhanced $^{13}$C-$^{13}$C refocused INADEQUATE spectrum of unlabeled plant samples collected 30-m inland. Signals are resolved for cellulose and xylan. (**B**) 2D Carbohydrate and aliphatic region of 2D $^{1}$H-$^{13}$C HETCOR spectrum of the 30-m inland plant. A short 0.1 ms CP was used to emphasize the one-bond correlations. (**C**) The aromatic region collected with long (1 ms) CP contact to show aromatic-aliphatic correlations. No cross peaks were observed with carbohydrates. The spectroscopic features are largely consistent in the plants collected at different locations of the island.

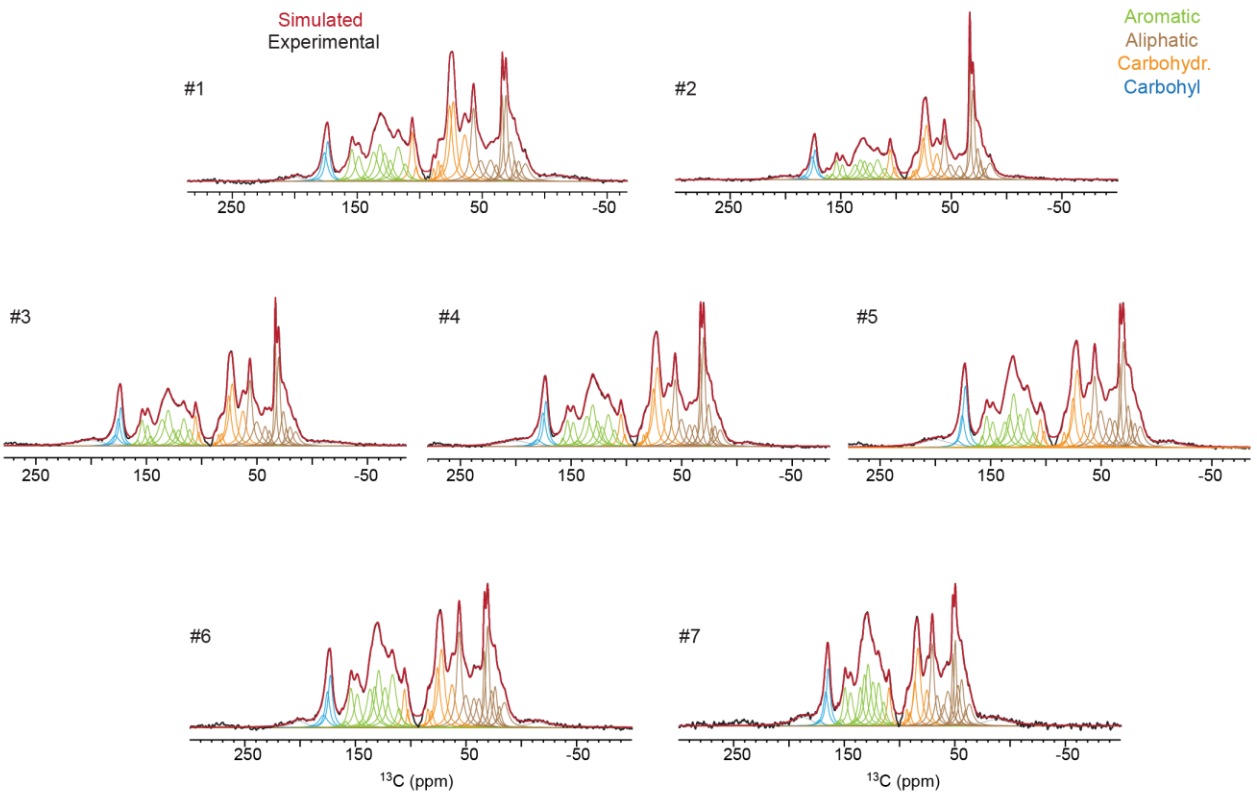


**Fig. S6. Spectral deconvolution of $^{13}$C MultiCP spectra for molecular composition.** For each sample, the simulated spectra (dark red) fit the experimentally measured 1D $^{13}$C MultiCP spectra (black). Underneath are the individual peaks that contribute to carbohydrate (orange), aliphatic (brown), aromatic (green) and carbonyl sites (blue). The peak list is guided by the resolvable sites obtained from high-resolution 2D data. Information on the deconvolution was documented in **Table S1**.

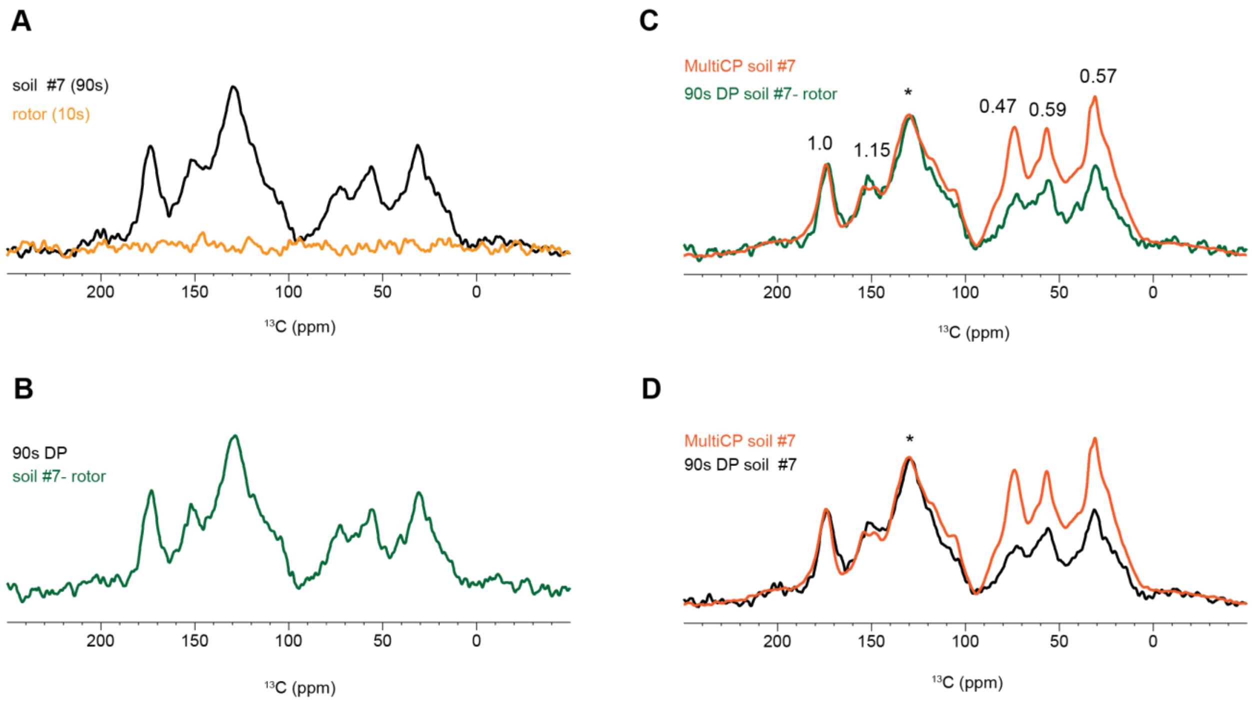


**Fig. S7. Comparison of 1D quantitative spectra acquired by MultiCP and Bloch decay experiment.** (**A**) 1D $^{13}$C Bloch decay (DP) spectra of soil sample 7 (black) and empty rotor (orange) measured with 90 second and 10 second recycle delays, respectively. Each spectrum was collected with 6,144 scans under 14 kHz MAS. (**B**) 1D quantitative difference spectra generated by subtraction of the rotor spectrum from the soil spectrum. (**C**) Overlay of MultiCP spectra (orange) with the quantitative difference spectra (green) normalized with respect to 130 ppm aromatic peak. Scaling factors have been obtained to account for the preferential detection of carbohydrate and aliphatic signals in MultiCP spectrum. (**D**) Overlay of MultiCP spectrum (orange) with the quantitative DP spectrum (black) of the soil sample 7 normalized with respect to the 130 ppm peak (asterisk).

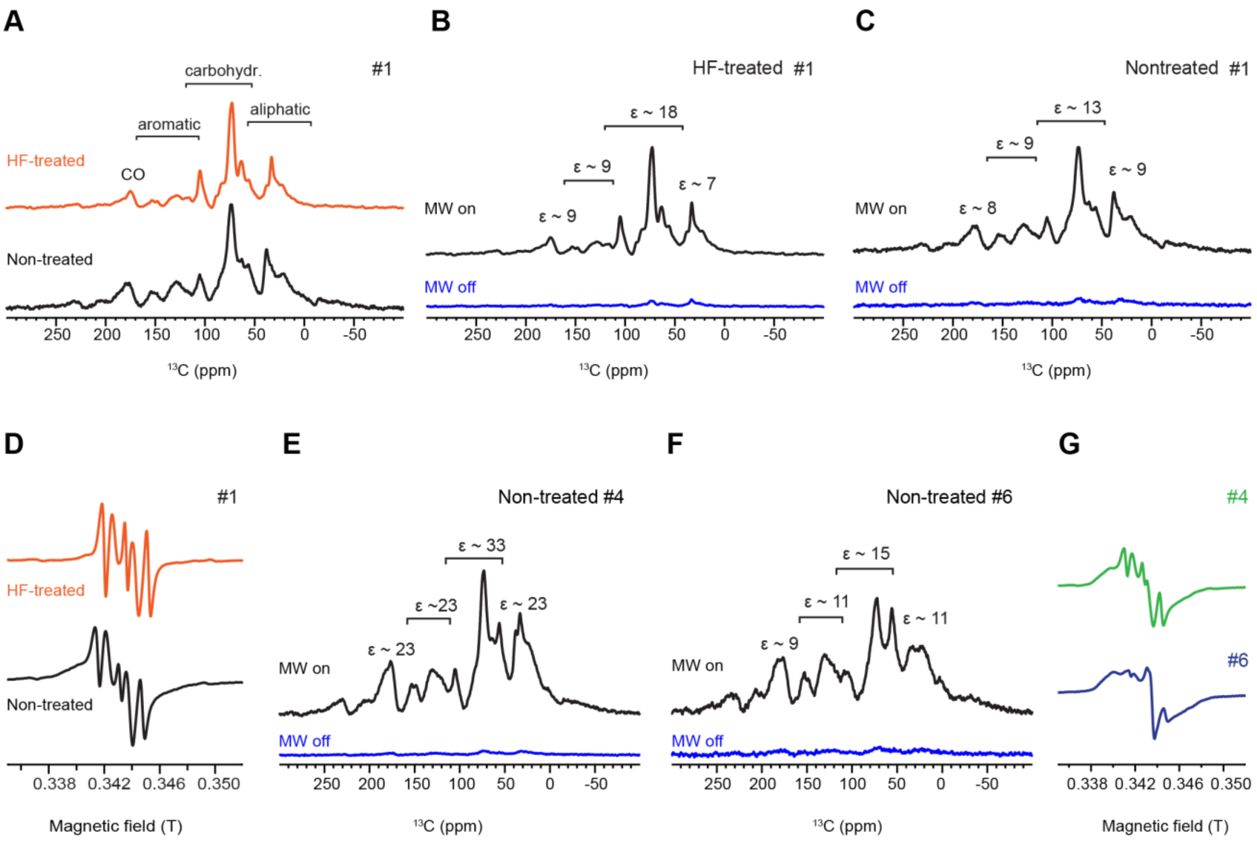


**Fig. S8. 1D DNP $^{13}$C spectra and EPR of soil samples.** (**A**) Comparison of $^{13}$C spectra of the HF and non-HF treated materials of soil sample #1 under DNP enhancement. (**B**) Comparison of $^{13}$C spectra with and without microwave (MW) irradiation collected on HF-treated soil sample 1. The enhancement is 18-fold for carbohydrates, 9-fold for aromatics, 7-fold for CO, 9-fold for most aliphatic carbons, and 7-fold for the polymethylene $CH_2$ peak. (**C**) MW-on and MW-off spectra of non-treated soil sample 1. The enhancement is 13-fold for carbohydrates, 9-fold for aromatics, 8-fold for CO, and 8-fold for most aliphatic carbons, and 9-fold for the polymethylene $CH_2$ peak. (**D**) EPR spectra of soil sample #1 with (top) and without (bottom) HF treatment, hydrated using the $d_6$-DMSO/$D_2O$/$H_2O$ matrix. (**E**) $^{13}$C spectra with and without microwave (MW) irradiation collected on native soil sample #4 without HF treatment, showing enhancement factor of 33-fold for carbohydrate and 23-fold for all other carbon sites. (**F**) MW-on and MW-off spectra of untreated soil sample #6, showing enhancement of 15 for carbohydrate and 9-11 for other carbon sites. (**G**) EPR spectra of AsymPolPOK at 9.6 GHz for untreated sample #4 (green) and #6 (blue), with a solvent of $d_6$-DMSO/$D_2O$ (90:10 Vol%).

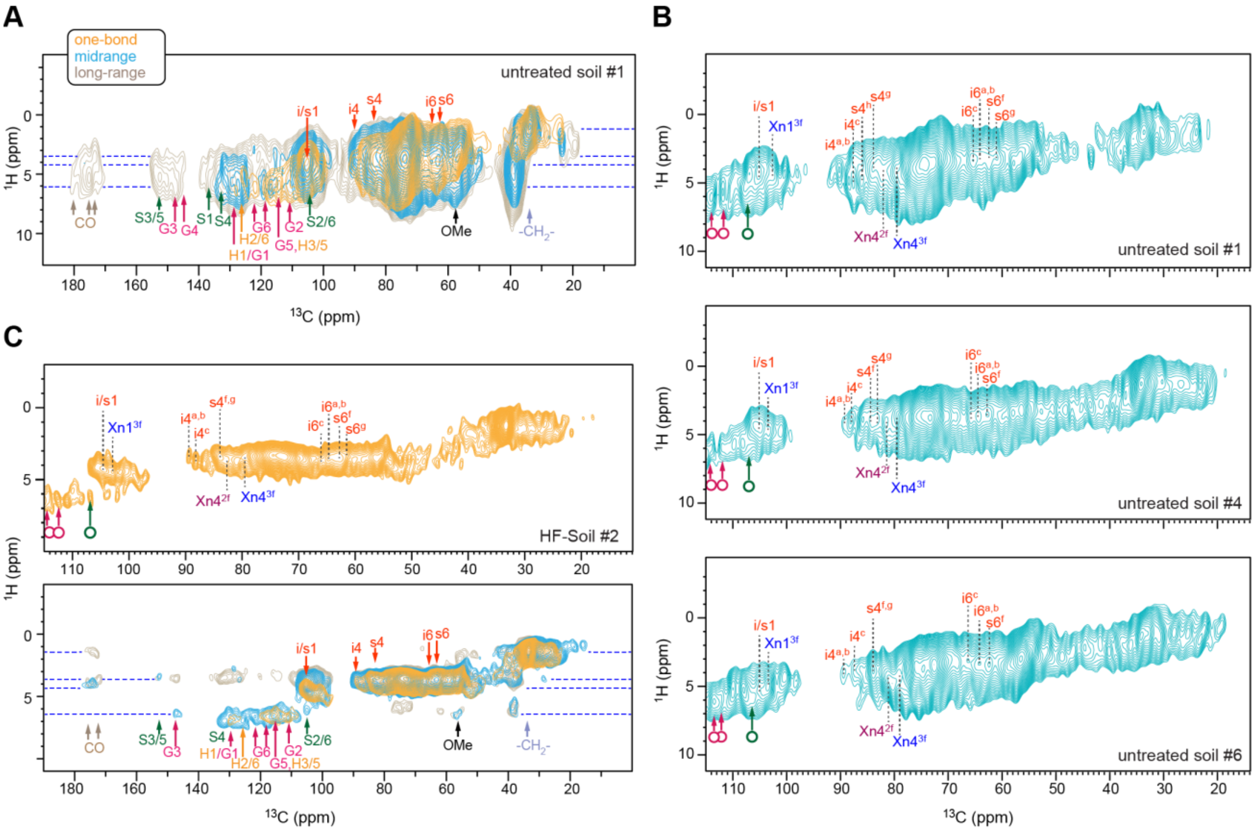


**Fig. S9. 2D $^{1}$H-$^{13}$C correlation DNP spectra of untreated soil.** (**A**) 2D $^{1}$H-$^{13}$C correlation spectra of untreated soil sample 1 measured with 0.1 ms (yellow), 0.5 ms (blue), and 1 ms (grey) CP contact times. (**B**) Zoom-in regions of carbohydrate and aliphatic signals in three untreated soil samples (1, 4, and 6). (**C**) Additional 2D $^{1}$H-$^{13}$C spectra of HF-treated soil sample 2. The key signals of carbohydrates and aromatics are observable. Top and bottom panels show the aliphatic/carbohydrate and aromatic signals, respectively.

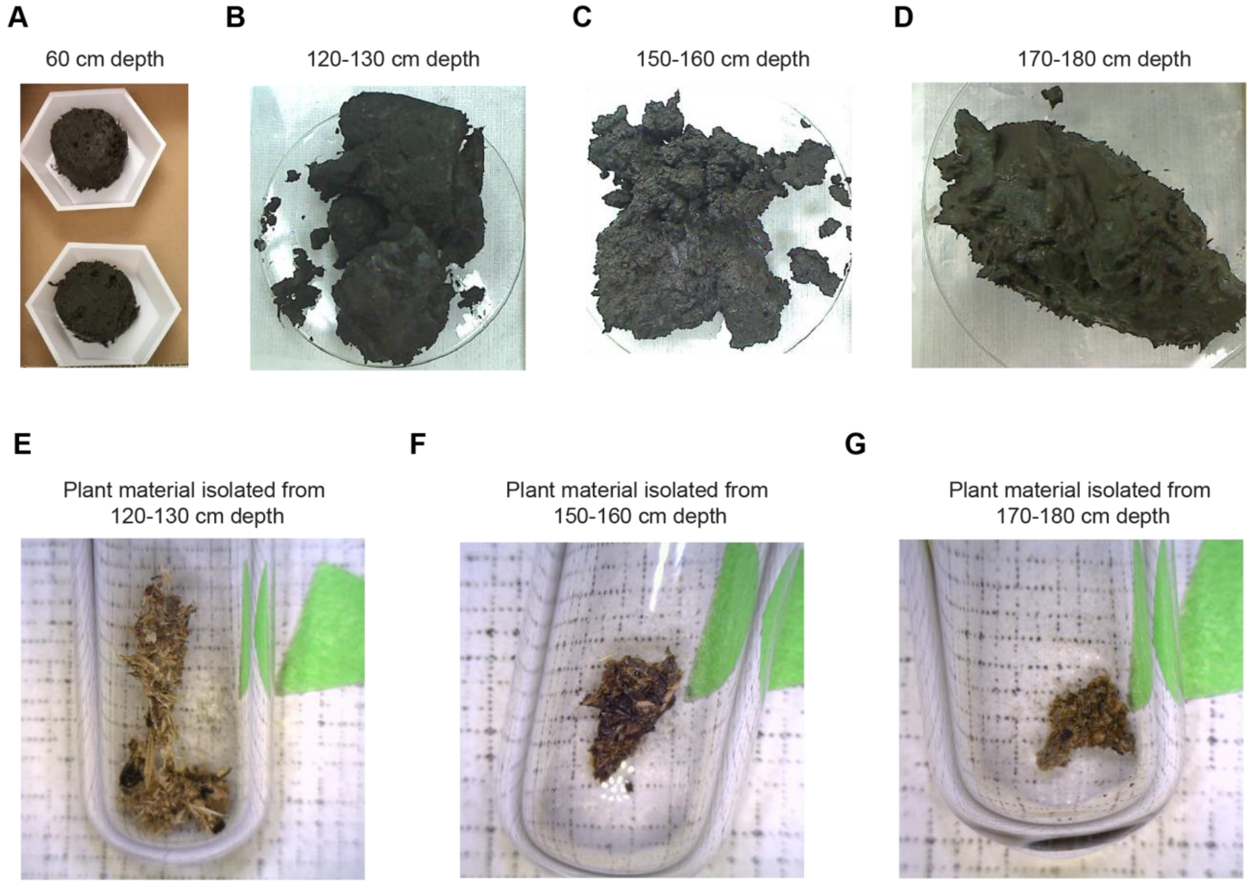


**Fig. S10. Isolation of plant materials from soil samples across the full range of depth.** Pictures of soil materials extracted at depths of (**A**) 60 cm, (**B**) 120-130 cm, (**C**) 150-160 cm, and (**D**) 170-180 cm. Fibril-like segments can be observed in these samples. Pictures are also shown for the plant materials isolated from the soil samples at depths of (**E**) 120-130 cm, (**F**) 150-160 cm, and (**G**) 170-180 cm.

**Table S1. Deconvolution parameters of MultiCP $^{13}C$ spectra for HF-treated soil samples before calibration.** The attributed chemical shifts (CSs) are labeled. The integral value (I), linewidth (L), and relative amplitude (A) are listed. Uncertain (U).

| | **Assign.** | **Soil 1** | **Soil 2** | **Soil 3** | **Soil 4** | **Soil 5** | **Soil 6** | **Soil 7** |
|---|---|---|---|---|---|---|---|---|
| | | CSs(ppm); I(%) [L(ppm), A(%)] | CSs(ppm); I(%) [L(ppm), A(%)] | CSs(ppm); I(%) [L(ppm), A(%)] | CSs(ppm); I(%) [L(ppm), A(%)] | CSs(ppm); I(%) [L(ppm), A(%)] | CSs(ppm); I(%) [L(ppm), A(%)] | CSs(ppm); I(%) [L(ppm), A(%)] |
| Carbonyl | CO | 182.3; 0.29 [6.5, 0.2] | 183.8; 0.47 [4.1, 0.5] | 178.7; 2.02 [8.7, 1.2] | 181.0; 1.21 [9.6, 0.7] | 180.2; 0.97 [9.0, 0.6] | 179.4; 1.68 [9.2, 1.0] | 183.2; 0.70 [8.0, 0.5] |
| | CO | 176.1; 3.16 [5.7, 2.8] | 176.2; 3.86 [6.2, 3.0] | 175.8; 2.96 [4.9, 3.0] | 175.7; 3.55 [5.6, 3.6] | 176.1; 2.84 [5.3, 3.0] | 175.7; 2.65 [4.9, 3.0] | 176.2; 3.19 [6.3, 3.0] |
| | CO | 173.4; 3.40 [4.4, 3.9] | 173.6; 3.53 [4.3, 3.9] | 173.5; 3.63 [4.2, 4.3] | 173.1; 3.92 [4.6, 4.5] | 173.3; 5.18 [5.1, 5.7] | 173.1; 3.72 [4.8, 4.4] | 173.3; 4.47 [5.3, 5.1] |
| Aromatic | H4 | 161.5; 0.17 [2.4, 0.4] | 162.8; 0.41 [2.9, 0.8] | 161.6; 0.07 [1.5, 0.2] | 161.5; 0.08 [2.3, 0.2] | 161.1; 0.08 [1.5, 0.3] | 162.7; 0.06 [1.1, 0.3] | 162.3; 0.13 [1.6, 0.5] |
| | S3/5[b] | 158.4; 0.24 [3.1, 0.4] | 158.1; 0.70 [4.5, 0.7] | 158.4; 0.38 [0.7, 2.9] | 157.8; 0.63 [3.9, 0.9] | 157.8; 0.69 [3.5, 1.1] | 159.0; 0.44 [4.7, 0.5] | 158.5; 0.46 [4.7, 0.6] |
| | S3/5[a] | 154.1; 3.17 [5.2, 3.1] | 154.2; 2.30 [4.4, 2.5] | 154.2; 2.75 [4.8, 2.9] | 153.6; 2.44 [5.0, 2.6] | 153.9; 2.69 [5.2, 2.9] | 154.4; 3.21 [5.5, 3.3] | 154.4; 3.14 [5.5, 3.4] |
| | G3 | 148.7; 3.12 [6.5, 2.4] | 148.9; 1.89 [4.6, 1.9] | 149.4; 2.18 [4.7, 0.3] | 148.4; 2.60 [5.8, 2.4] | 148.5; 2.54 [6.0, 2.4] | 148.8; 3.18 [6.4, 2.8] | 148.3; 3.55 [7.2, 2.9] |
| | G4 | 144.4; 0.17 [2.0, 0.4] | 146.0; 0.51 [4.0, 0.6] | 146.6; 1.19 [5.3, 1.1] | 144.5; 0.66 [6.8, 0.5] | 144.6; 0.86 [5.6, 0.9] | 145.0; 0.41 [4.2, 0.5] | 141.8; 0.92 [9.1, 0.6] |
| | S1 | 136.6; 4.43 [7.9, 2.8] | 137.4; 3.07 [7.5, 2.0] | 136.2; 5.40 [9.2, 2.9] | 136.1; 5.04 [9.0,3.0] | 137.5; 3.20 [7.6, 2.4] | 137.1; 4.38 [7.6, 3.2] | 137.2; 3.70 [6.4, 3.4] |
| | G1/H1 | 131.7; 4.79 [6.8, 3.6] | 132.6; 4.04 [7.5, 2.6] | 130.7; 6.24 [7.9, 3.9] | 130.9; 5.90 [7.6, 4.2] | 133.4; 3.74 [7.0, 3.0] | 133.0; 3.83 [6.3, 3.4] | 132.6; 4.99 [6.6, 4.5] |
| | H2/6 | 127.8; 3.64 [6.7, 2.8] | 128.9; 3.30 [6.5, 2.4] | 125.8; 2.43 [6.7, 1.8] | 126.5; 3.47 [7.4, 2.5] | 129.6; 6.57 [7.4, 5.0] | 129.4; 6.07 [7.1, 4.7] | 128.7; 6.77 [7.4, 5.4] |
| | G6 | 123.4; 2.59 [6.4, 2.0] | 123.9; 3.57 [7.9, 2.6] | 121.4; 2.41 [6.8, 1.8] | 122.3; 2.47 [6.7, 2.0] | 123.8; 4.59 [8.4, 3.1] | 123.8; 4.63 [7.8, 3.3] | 123.1; 5.29 [8.2, 3.9] |
| | G5, H3/5 | 117.0; 4.67 [7.4, 3.4] | 117.1; 4.12 [7.9, 2.6] | 116.6; 3.74 [6.5, 3.0] | 116.7; 4.13 [7.3, 3.2] | 117.0; 4.65 [7.6, 3.6] | 116.9; 6.11 [8.1, 4.4] | 116.6; 4.42 [7.3, 3.8] |
| | G2 | 111.4; 2.02 [6.1, 1.7] | 110.8; 1.95 [6.3, 1.5] | 111.4; 2.06 [5.6, 1.8] | 111.2; 1.90 [6.2, 1.6] | 111.3; 1.66 [6.6, 1.4] | 111.0; 1.67 [5.9, 1.6] | 111.0; 2.30 [6.5, 2.1] |
| Carbohydrate | i/s1 | 105.9; 3.71 [3.9, 4.8] | 105.7; 3.57 [4.3, 4.0] | 106.0; 2.52 [3.7, 3.4] | 105.3; 2.65 [4.4, 3.2] | 105.5; 2.05 [4.4, 2.6] | 106.1; 2.27 [4.0, 3.2] | 105.2; 2.68 [4.7, 3.4] |
| | Xn[3f]1 | 102.7; 0.89 [3.0, 1.5] | 101.9; 1.20 [3.5, 1.6] | 103.1; 0.96 [3.1, 1.6] | 101.9; 0.74 [3.2, 1.2] | 102.3; 0.88 [3.2, 1.5] | 103.1; 0.73 [2.7, 1.5] | 101.0; 0.08 [1.2, 0.4] |
| | i4 | 88.8; 0.48 [2.3, 1.3] | 88.2; 0.07 [1.4, 0.3] | 88.5; 0.12 [1.8, 0.4] | 88.4; 0.11 [1.6, 0.4] | 88.6; 0.01 [0.5, 0.1] | 88.6; 0.03 [0.9, 0.2] | 88.5; 0.03 [0.8, 0.3] |
| | s4 | 84.9; 1.18 [3.0, 2.0] | 84.9; 0.64 [2.6, 1.2] | 85.1; 0.71 [2.7, 1.3] | 84.7; 0.63 [2.8, 1.2] | 84.0; 0.86 [3.4, 1.4] | 85.0; 0.83 [3.0, 1.6] | 85.0; 0.78 [3.1, 1.5] |
| | Xn[2f]4 | 82.4; 1.26 [4.0, 1.6] | 82.6; 1.01 [3.7, 1.3] | 82.4; 1.04 [3.7, 1.4] | 82.2; 0.95 [3.6, 1.4] | 80.2; 1.02 [4.2, 1.3] | 81.7; 1.06 [4.2, 1.4] | 81.8; 0.87 [4.3, 1.2] |
| | C2,3,5 | 75.9; 7.34 [5.0, 7.4] | 76.0; 6.14 [5.4, 5.4] | 76.0; 5.78 [5.2, 5.5] | 75.9; 6.09 [5.7, 5.7] | 75.8; 4.33 [5.3, 4.5] | 76.2; 4.88 [5.4, 5.0] | 76.2; 3.49 [5.3, 3.9] |
| | C2,3,5 | 73.0; 8.16 [5.3, 7.8] | 72.9; 9.88 [6.7, 7.0] | 72.9; 8.63 [6.2, 6.9] | 72.4; 10.2 [6.9, 7.9] | 72.1; 8.73 [6.8, 7.1] | 72.7; 7.72 [6.6, 6.5] | 72.8; 8.47 [7.4, 6.8] |
| | C6 | 63.8; 6.54 [7.4, 4.5] | 63.5; 4.75 [6.7, 3.3] | 63.2; 5.22 [6.7, 3.8] | 62.6; 5.02 [7.2, 3.7] | 62.5; 4.08 [7.1, 3.2] | 63.1; 4.64 [7.3, 3.6] | 62.7; 3.69 [6.9, 3.2] |
| Aliphatic | $-OCH_3$[b] or protein Cα | 56.8; 6.65 [4.7, 7.1] | 56.8; 5.48 [4.5, 5.7] | 56.8; 6.98 [4.8, 7.2] | 56.4; 6.19 [5.0, 6.6] | 56.4; 5.90 [5.0, 6.6] | 56.6; 7.32 [5.1, 8.0] | 56.6; 6.32 [5.2, 7.2] |
| | $-OCH_3$[a] or protein Cα | 51.1; 3.26 [8.2, 2.0] | 51.4; 3.38 [8.3, 2.0] | 50.8; 4.55 [8.5, 2.7] | 50.7; 3.86 [7.6, 2.7] | 51.0; 5.04 [8.4, 3.4] | 50.7; 4.02 [8.4, 2.7] | 51.0; 3.27 [7.3, 2.7] |

| | | | | | | | | |
|---|---|---|---|---|---|---|---|---|
| | $R_3CH$ & $R_4C$ | 43.1; 3.10 [7.6, 2.1] | 43.1; 2.41 [6.4, 1.8] | 43.1; 2.45 [5.6, 2.2] | 43.2; 2.51 [6.2, 2.2] | 42.8; 3.47 [7.2, 2.7] | 43.3; 2.87 [6.6, 2.4] | 43.9; 2.41 [7.8, 1.9] |
| | -CCH | 38.6; 1.51 [4.8, 1.6] | 39.3; 0.62 [3.2, 1.0] | 39.2; 1.44 [4.2, 1.7] | 38.6; 2.33 [5.9, 2.1] | 38.2; 2.90 [6.5, 2.5] | 38.9; 2.93 [6.8, 2.4] | 39.1; 4.55 [8.9, 3.0] |
| | $-(CH_2)_n-_a$ | 33.9; 3.64 [2.2, 8.5] | 33.9; 6.63 [1.9, 16.8] | 33.8; 4.74 [2.0, 12.0] | 33.5; 3.70 [2.1, 9.3] | 33.5; 3.12 [2.2, 7.7] | 33.9; 2.66 [2.3, 6.5] | 33.6; 2.77 [2.6, 6.4] |
| | $-(CH_2)_n-_b$ | 30.9; 5.63 [3.4, 8.4] | 31.0; 9.21 [3.7, 11.6] | 31.0; 7.26 [3.6, 9.8] | 30.6; 7.52 [3.7, 10.9] | 30.6; 7.04 [4.0, 9.8] | 31.0; 5.67 [3.7, 8.5] | 30.8; 4.30 [3.4, 7.5] |
| | -$\underline{CH_2}$-CH=CH- | 27.0; 4.81 [6.3, 3.9] | 26.6; 4.43 [5.2, 4.0] | 26.6; 4.14 [5.4, 3.8] | 26.1; 4.60 [5.9, 4.2] | 26.1; 3.92 [5.7, 3.9] | 27.3; 3.08 [5.6, 3.1] | 27.6; 3.73 [6.2, 3.6] |
| | $Ac^{Me}$ | 23.9; 1.32 [3.2, 2.1] | 24.0; 1.98 [3.6, 2.6] | 23.9; 1.31 [3.3, 2.0] | 23.6; 0.86 [2.5, 1.8] | 23.6; 1.63 [3.6, 2.5] | 24.1; 2.75 [4.5, 3.4] | 23.6; 4.25 [6.2, 4.1] |
| | $-CH_3$ | 20.6; 2.05 [5.3, 2.0] | 20.4; 1.14 [3.5, 1.5] | 20.5; 2.35 [5.5, 2.1] | 20.3; 1.82 [4.7, 2.0] | 20.0; 2.08 [5.2, 2.2] | 20.4; 1.40 [4.4, 1.8] | 19.3; 1.20 [5.1, 1.4] |
| | $-CH_3^U$ | 15.3; 2.63 [7.8, 1.7] | 15.5; 3.75 [8.4, 2.1] | 15.5; 2.35 [7.4, 1.6] | 15.6; 2.23 [7.2, 1.7] | 15.3; 2.70 [7.8, 1.9] | 15.8; 3.11 [8.4, 2.1] | 15.1; 3.06 [9.5, 1.9] |

**Table S2. Deconvolution parameters of MultiCP $^{13}C$ spectra for HF-treated soil samples after calibration using scaling factors obtained from quantitative DP spectra (Fig. S7).** The attributed chemical shifts (CSs) are labeled. The integral value (I), linewidth (L), and relative amplitude (A) are listed. Uncertain (U).

| | **Assign.** | **Soil 1** | **Soil 2** | **Soil 3** | **Soil 4** | **Soil 5** | **Soil 6** | **Soil 7** |
|---|---|---|---|---|---|---|---|---|
| | | CSs(ppm); I(%) [L(ppm), A(%)] | CSs(ppm); I(%) [L(ppm), A(%)] | CSs(ppm); I(%) [L(ppm), A(%)] | CSs(ppm); I(%) [L(ppm), A(%)] | CSs(ppm); I(%) [L(ppm), A(%)] | CSs(ppm); I(%) [L(ppm), A(%)] | CSs(ppm); I(%) [L(ppm), A(%)] |
| Carbonyl | CO | 182.3; 0.39 [6.5, 0.2] | 183.8; 0.65 [4.1, 0.5] | 178.7; 2.72 [8.7, 1.2] | 181.0; 1.64 [9.6, 0.7] | 180.2; 1.29 [9.0, 0.6] | 179.4; 2.21 [9.2, 1.0] | 183.2; 0.91 [8.0, 0.5] |
| | CO | 176.1; 4.28 [5.7, 2.8] | 176.2; 5.30 [6.2, 3.0] | 175.8; 3.99 [4.9, 3.0] | 175.7; 4.78 [5.6, 3.6] | 176.1; 3.78 [5.3, 3.0] | 175.7; 3.48 [4.9, 3.0] | 176.2; 4.14 [6.3, 3.0] |
| | CO | 173.4; 4.60 [4.4, 3.9] | 173.6; 4.84 [4.3, 3.9] | 173.5; 4.89 [4.2, 4.3] | 173.1; 5.28 [4.6, 4.5] | 173.3; 6.88 [5.1, 5.7] | 173.1; 4.87 [4.8, 4.4] | 173.3; 5.80 [5.3, 5.1] |
| Aromatic | H4 | 161.5; 0.26 [2.4, 0.4] | 162.8; 0.64 [2.9, 0.8] | 161.6; 0.11 [1.5, 0.2] | 161.5; 0.12 [2.3, 0.2] | 161.1; 0.12 [1.5, 0.3] | 162.7; 0.09 [1.1, 0.3] | 162.3; 0.20 [1.6, 0.5] |
| | S3/5[b] | 158.4; 0.37 [3.1, 0.4] | 158.1; 1.11 [4.5, 0.7] | 158.4; 0.60 [0.7, 2.9] | 157.8; 0.98 [3.9, 0.9] | 157.8; 1.06 [3.5, 1.1] | 159.0; 0.67 [4.7, 0.5] | 158.5; 0.69 [4.7, 0.6] |
| | S3/5[a] | 154.1; 4.93 [5.2, 3.1] | 154.2; 3.64 [4.4, 2.5] | 154.2; 4.27 [4.8, 2.9] | 153.6;3.78 [5.0, 2.6] | 153.9; 4.11 [5.2, 2.9] | 154.4; 4.84 [5.5, 3.3] | 154.4; 4.68 [5.5, 3.4] |
| | G3 | 148.7; 4.86 [6.5, 2.4] | 148.9; 2.98 [4.6, 1.9] | 149.4; 3.38 [4.7, 0.3] | 148.4; 4.03 [5.8, 2.4] | 148.5; 3.89 [6.0, 2.4] | 148.8; 4.79 [6.4, 2.8] | 148.3; 5.29 [7.2, 2.9] |
| | G4 | 144.4; 0.24 [2.0, 0.4] | 146.0; 0.70 [4.0, 0.6] | 146.6; 1.61 [5.3, 1.1] | 144.5; 0.89 [6.8, 0.5] | 144.6; 1.14 [5.6, 0.9] | 145.0; 0.54 [4.2, 0.5] | 141.8; 1.20 [9.1, 0.6] |
| | S1 | 136.6; 5.99 [7.9, 2.8] | 137.4; 4.22 [7.5, 2.0] | 136.2; 7.28 [9.2, 2.9] | 136.1; 6.80 [9.0,3.0] | 137.5; 4.25 [7.6, 2.4] | 137.1; 5.74 [7.6, 3.2] | 137.2; 4.80 [6.4, 3.4] |
| | G1/H1 | 131.7; 6.48 [6.8, 3.6] | 132.6; 5.54 [7.5, 2.6] | 130.7; 8.42 [7.9, 3.9] | 130.9; 7.95 [7.6, 4.2] | 133.4; 4.97 [7.0, 3.0] | 133.0; 5.02 [6.3, 3.4] | 132.6; 6.47 [6.6, 4.5] |
| | H2/6 | 127.8; 4.92 [6.7, 2.8] | 128.9; 4.52 [6.5, 2.4] | 125.8; 3.28 [6.7, 1.8] | 126.5; 4.67 [7.4, 2.5] | 129.6; 8.72 [7.4, 5.0] | 129.4; 7.96 [7.1, 4.7] | 128.7; 8.78 [7.4, 5.4] |
| | G6 | 123.4; 3.51 [6.4, 2.0] | 123.9; 4.90 [7.9, 2.6] | 121.4; 3.26 [6.8, 1.8] | 122.3; 3.34 [6.7, 2.0] | 123.8; 6.09 [8.4, 3.1] | 123.8; 6.07 [7.8, 3.3] | 123.1; 6.86 [8.2, 3.9] |
| | G5, H3/5 | 117.0; 6.32 [7.4, 3.4] | 117.1; 5.65 [7.9, 2.6] | 116.6; 5.05 [6.5, 3.0] | 116.7; 5.56 [7.3, 3.2] | 117.0; 6.17 [7.6, 3.6] | 116.9; 8.01 [8.1, 4.4] | 116.6; 5.73 [7.3, 3.8] |
| | G2 | 111.4; 2.73 [6.1, 1.7] | 110.8; 2.68 [6.3, 1.5] | 111.4; 2.78 [5.6, 1.8] | 111.2; 2.57 [6.2, 1.6] | 111.3; 2.21 [6.6, 1.4] | 111.0; 2.19 [5.9, 1.6] | 111.0; 2.99 [6.5, 2.1] |
| Carbohydrate | i/s1 | 105.9; 5.02 [3.9, 4.8] | 105.7; 4.90 [4.3, 4.0] | 106.0; 3.40 [3.7, 3.4] | 105.3; 3.58 [4.4, 3.2] | 105.5; 2.72 [4.4, 2.6] | 106.1; 2.97 [4.0, 3.2] | 105.2; 3.47 [4.7, 3.4] |
| | $Xn^{3f}1$ | 102.7; 1.20 [3.0, 1.5] | 101.9; 1.65 [3.5, 1.6] | 103.1; 1.30 [3.1, 1.6] | 101.9; 0.99 [3.2, 1.2] | 102.3; 1.16 [3.2, 1.5] | 103.1; 0.96 [2.7, 1.5] | 101.0; 0.11 [1.2, 0.4] |
| | i4 | 88.8; 0.30 [2.3, 1.3] | 88.2; 0.05 [1.4, 0.3] | 88.5; 0.08 [1.8, 0.4] | 88.4; 0.07 [1.6, 0.4] | 88.6; 0.01 [0.5, 0.1] | 88.6; 0.02 [0.9, 0.2] | 88.5; 0.02 [0.8, 0.3] |
| | s4 | 84.9; 0.75 [3.0, 2.0] | 84.9; 0.41 [2.6, 1.2] | 85.1; 0.45 [2.7, 1.3] | 84.7; 0.40 [2.8, 1.2] | 84.0; 0.54 [3.4, 1.4] | 85.0; 0.51 [3.0, 1.6] | 85.0; 0.47 [3.1, 1.5] |
| | $Xn^{2f}4$ | 82.4; 0.80 [4.0, 1.6] | 82.6; 0.65 [3.7, 1.3] | 82.4; 0.66 [3.7, 1.4] | 82.2; 0.60 [3.6, 1.4] | 80.2; 0.64 [4.2, 1.3] | 81.7; 0.65 [4.2, 1.4] | 81.8; 0.53 [4.3, 1.2] |
| | C2,3,5 | 75.9; 4.67 [5.0, 7.4] | 76.0; 3.96 [5.4, 5.4] | 76.0; 3.66 [5.2, 5.5] | 75.9; 3.86 [5.7, 5.7] | 75.8; 2.70 [5.3, 4.5] | 76.2; 3.01 [5.4, 5.0] | 76.2; 2.13 [5.3, 3.9] |
| | C2,3,5 | 73.0; 5.19 [5.3, 7.8] | 72.9; 6.38 [6.7, 7.0] | 72.9; 5.47 [6.2, 6.9] | 72.4; 6.46 [6.9, 7.9] | 72.1; 5.45 [6.8, 7.1] | 72.7; 4.76 [6.6, 6.5] | 72.8; 5.16 [7.4, 6.8] |
| | C6 | 63.8; 5.22 [7.4, 4.5] | 63.5; 3.85 [6.7, 3.3] | 63.2; 4.15 [6.7, 3.8] | 62.6; 3.99 [7.2, 3.7] | 62.5; 3.20 [7.1, 3.2] | 63.1; 3.59 [7.3, 3.6] | 62.7; 2.82 [6.9, 3.2] |
| Aliphatic | $-OCH_3$[b] or protein Cα | 56.8; 5.31 [4.7, 7.1] | 56.8; 4.44 [4.5, 5.7] | 56.8; 5.56 [4.8, 7.2] | 56.4; 4.92 [5.0, 6.6] | 56.4; 4.62 [5.0, 6.6] | 56.6; 5.66 [5.1, 8.0] | 56.6; 4.84 [5.2, 7.2] |
| | $-OCH_3$[a] or | 51.1; 2.60 [8.2, 2.0] | 51.4; 2.73 [8.3, 2.0] | 50.8; 3.62 [8.5, 2.7] | 50.7; 3.07 [7.6, 2.7] | 51.0; 3.95 [8.4, 3.4] | 50.7; 3.11 [8.4, 2.7] | 51.0; 2.50 [7.3, 2.7] |

| | | | | | | | | |
|---|---|---|---|---|---|---|---|---|
| | protein Cα | | | | | | | |
| | $R_3CH$ & $R_4C$ | 43.1; 2.39 [7.6, 2.1] | 43.1; 1.88 [6.4, 1.8] | 43.1; 1.88 [5.6, 2.2] | 43.2; 1.93 [6.2, 2.2] | 42.8; 2.63 [7.2, 2.7] | 43.3; 2.14 [6.6, 2.4] | 43.9; 1.78 [7.8, 1.9] |
| | -CCH | 38.6; 1.17 [4.8, 1.6] | 39.3; 0.49 [3.2, 1.0] | 39.2; 1.11 [4.2, 1.7] | 38.6; 1.79 [5.9, 2.1] | 38.2; 2.19 [6.5, 2.5] | 38.9; 2.19 [6.8, 2.4] | 39.1; 3.36 [8.9, 3.0] |
| | $-(CH_2)_n-_a$ | 33.9; 2.81 [2.2, 8.5] | 33.9; 5.19 [1.9, 16.8] | 33.8; 3.65 [2.0, 12.0] | 33.5; 2.84 [2.1, 9.3] | 33.5; 2.36 [2.2, 7.7] | 33.9; 1.99 [2.3, 6.5] | 33.6; 2.05 [2.6, 6.4] |
| | $-(CH_2)_n-_b$ | 30.9; 4.34 [3.4, 8.4] | 31.0; 7.21 [3.7, 11.6] | 31.0; 5.58 [3.6, 9.8] | 30.6; 5.78 [3.7, 10.9] | 30.6; 5.33 [4.0, 9.8] | 31.0; 4.24 [3.7, 8.5] | 30.8; 3.18 [3.4, 7.5] |
| | $-\underline{CH_2}-CH=CH-$ | 27.0; 3.71 [6.3, 3.9] | 26.6; 3.46 [5.2, 4.0] | 26.6; 3.18 [5.4, 3.8] | 26.1; 3.53 [5.9, 4.2] | 26.1; 2.97 [5.7, 3.9] | 27.3; 2.30 [5.6, 3.1] | 27.6; 2.76 [6.2, 3.6] |
| | $Ac^{Me}$ | 23.9; 1.02 [3.2, 2.1] | 24.0; 1.55 [3.6, 2.6] | 23.9; 1.01 [3.3, 2.0] | 23.6; 0.66 [2.5, 1.8] | 23.6; 1.23 [3.6, 2.5] | 24.1; 2.05 [4.5, 3.4] | 23.6; 3.14 [6.2, 4.1] |
| | $-CH_3$ | 20.6; 1.58 [5.3, 2.0] | 20.4; 0.89 [3.5, 1.5] | 20.5; 1.81 [5.5, 2.1] | 20.3; 1.40 [4.7, 2.0] | 20.0; 1.58 [5.2, 2.2] | 20.4; 1.05 [4.4, 1.8] | 19.3; 0.89 [5.1, 1.4] |
| | $-CH_3^U$ | 15.3; 2.03 [7.8, 1.7] | 15.5; 2.93 [8.4, 2.1] | 15.5; 1.81 [7.4, 1.6] | 15.6; 1.71 [7.2, 1.7] | 15.3; 2.04 [7.8, 1.9] | 15.8; 2.33 [8.4, 2.1] | 15.1; 2.26 [9.5, 1.9] |

**Table S3. Molecular composition of HF-treated soils based on spectral deconvolution of MultiCP spectra, with calibration by quantitative DP spectra**.

| Soil # | Depth (cm) | Carbohyd. | Aromatic | Aliphatic | CO | i4/s4 [a] | S/G [b] | [c]-$(CH_2)_n$- | [d]a/b | -$OCH_3$ | Era |
|---|---|---|---|---|---|---|---|---|---|---|---|
| 1 | 0-10 | 23.2% | 40.6% | 27.0% | 9.3% | 0.40 | 1.04 | 7.2% | 0.65 | 7.9% | 2018 AD |
| 2 | 40-50 | 21.8% | 36.6% | 30.8% | 10.8% | 0.11 | 1.29 | 12.4% | 0.72 | 7.2% | 1963 AD |
| 3 | 80-90 | 19.2% | 40.0% | 29.2% | 11.6% | 0.17 | 0.97 | 9.2% | 0.65 | 9.2% | 1562 AD |
| 4 | 100-110 | 20.0% | 40.7% | 27.6% | 11.7% | 0.17 | 0.97 | 8.6% | 0.49 | 8.0% | 1169 AD |
| 5 | 130-140 | 16.4% | 42.7% | 28.9% | 11.9% | 0.01 | 1.03 | 7.7% | 0.44 | 8.6% | |
| 6 | 160-170 | 16.5% | 45.9% | 27.1% | 10.6% | 0.03 | 1.03 | 6.2% | 0.47 | 8.8% | 945 AD |
| 7 | 170-180 | 14.7% | 47.7% | 26.8% | 10.9% | 0.04 | 0.83 | 5.2% | 0.65 | 7.3% | |

[a] The ratio of interior cellulose to surface cellulose. Using integral of i4 peak over integrals of s4 peak.

[b] Lignin aromatic ring S unit to G unit ratio. Using integrals of S3/5 peaks over integrals of G3/4 peaks.

[c] Summation of integrals of aliphatic polymethylene -$(CH_2)_n$-[a] at 33 ppm and -$(CH_2)_n$-[b] at 31 ppm.

[d] Ratio of integrals of aliphatic polymethylene -$(CH_2)_n$-[a] at 33 ppm and -$(CH_2)_n$-[b] at 31 ppm.

**Table S4. Parameters of ssNMR experiments measured for soil and plant samples.** HF-treated soil samples (Soil #$^{HF}$); Non-treated soil samples (Soil #$^{Non}$); NS: number of scans; number of points of time domain for the direct (td2) and indirect (td1) dimensions; the acquisition time of the direct dimension (aq2); the evolution time of indirect dimension (aq1); d1: recycle delay. N/A: not applicable.

| | Sample | DNP juice | Experiment | Time | Figure # | MAS (kHz) | NS | td2 | td1 | aq2 (ms) | aq1 (ms) | d1 (s) |
|---|---|---|---|---|---|---|---|---|---|---|---|---|
| ssNMR 9.4 T 298 K | #1-7$^{HF}$ | N/A | $^{13}$C-MultiCP | 35 h | 2a, 4a, S6 | | 16k | 2494 | | 24.9 | | 1 |
| | #1$^{Non}$ | N/A | $^{13}$C-MultiCP | 35 h | 2a | 14 | 16k | 2494 | N/A | 41 | N/A | 2 |
| | #1$^{HF}$ | N/A | $^{13}$C-CP | 9.5 h | S3a | | 16k | 4096 | | 41 | | 2 |
| | #1-7$^{HF}$ | N/A | $^{13}$C-NQS | 9.5 h | S3 | | 16k/8k | 4096 | | 41 | | 2 |
| MAS DNP 14.1 T 100 K | #1$^{HF}$ | $D_2O/H_2O$, 9/1 | $^{13}$C-CP | 2 min | 2b, 3a, S8a, S8b | 8 | 64 | 4096 | | 22.9 | | 1.8 |
| | #1$^{Non}$ | d$_6$-DMSO/$H_2O$, 9/1 | $^{13}$C-CP | 0.6 h | S8a, S8c | 8 | 1k | 2048 | | 10.2 | | 2.1 |
| | #4$^{Non}$ | d$_6$-DMSO/$H_2O$, 9/1 | $^{13}$C-CP | 5 min | S8e | 8 | 128 | 2048 | N/A | 10.2 | N/A | 2.0 |
| | #6$^{Non}$ | d$_6$-DMSO/$H_2O$, 9/1 | $^{13}$C-CP | 0.25 h | S8f | 8 | 512 | 2048 | | 10.2 | | 1.7 |
| | plant (edge) | $D_2O/H_2O$, 9/1 | $^{13}$C-CP | 0.5 h | 3a, S4a, S4b | 10.5 | 512 | 4096 | | 22.9 | | 3 |
| | plant (inland) | $D_2O/H_2O$, 9/1 | $^{13}$C-CP | 0.75 h | S4c, S4d | 10.5 | 512 | 4096 | | 22.9 | | 5.2 |
| | #1$^{HF}$ | $D_2O/H_2O$, 9/1 | $^{13}$C dipolar-INADEQUATE | 16 h | 2c | 10.5 | 320 | 2048 | 98 | 17.2 | 2.7 | 1.8 |
| | plant (edge) | $D_2O/H_2O$, 9/1 | $^{13}$C dipolar-INADEQUATE | 13 h | 3b, 3c | 8 | 160 | 2048 | 100 | 17.2 | 1.7 | 2.9 |
| | plant (inland) | $D_2O/H_2O$, 9/1 | $^{13}$C dipolar-INADEQUATE | 23 h | S5a | 10.5 | 160 | 2048 | 100 | 17.2 | 1.7 | 5.2 |
| | #1$^{HF}$ | $D_2O/H_2O$, 9/1 | $^{1}$H-$^{13}$C HETCOR (PMLG) | 1 h | 2e | 8 | 40 | 4096 | 38 | 22.9 | 3.8 | 1.8 |
| | #1$^{HF}$ | d$_6$-DMSO/$D_2O/H_2O$ | $^{1}$H-$^{13}$C HETCOR (FSLG) | 4.5 h | 2g, S2 | 10.5 | 96 | 4096 | 64 | 22.9 | 2.4 | 2.6 |
| | #1$^{Non}$ | d$_6$-DMSO/$H_2O$, 9/1 | $^{1}$H-$^{13}$C HETCOR (FSLG) | 12 h | S9a, S9b | 8 | 512 | 2048 | 40 | 10.2 | 1.5 | 2.1 |
| | #1$^{Non}$ | d$_6$-DMSO/$H_2O$, 9/1 | $^{1}$H-$^{13}$C HETCOR (FSLG) | 24 h | S9a | 8 | 1k | 2048 | 40 | 10.2 | 1.5 | 2.1 |
| | #2$^{HF}$ | $D_2O/H_2O$, 9/1 | $^{1}$H-$^{13}$C HETCOR (FSLG) | 3 h | S9b | 10.5 | 32 | 4096 | 128 | 22.9 | 4.8 | 2.6 |
| | #4$^{Non}$ | d$_6$-DMSO/$H_2O$, 9/1 | $^{1}$H-$^{13}$C HETCOR (FSLG) | 1.4 h | 4c, S9b | 8 | 64 | 2048 | 40 | 10.2 | 1.5 | 2.0 |
| | #6$^{Non}$ | d$_6$-DMSO/$H_2O$, 9/1 | $^{1}$H-$^{13}$C HETCOR (FSLG) | 10 h | 4c, S9b | 8 | 512 | 2048 | 40 | 10.2 | 1.5 | 1.7 |
| | #6$^{Non}$ | d$_6$-DMSO/$H_2O$, 9/1 | $^{1}$H-$^{13}$C HETCOR (FSLG) | 7.3 h | 4c | 8 | 512 | 2048 | 30 | 10.2 | 1.1 | 1.7 |
| | plant (edge) | $D_2O/H_2O$, 9/1 | $^{1}$H-$^{13}$C HETCOR (FSLG) | 1.7 h | 3d, 3e | 10.5 | 8 | 4096 | 128 | 22.9 | 4.8 | 5.8 |
| | plant (inland) | $D_2O/H_2O$, 9/1 | $^{1}$H-$^{13}$C HETCOR (FSLG) | 1.7 h | S5b, S5c | 10.5 | 8 | 4096 | 128 | 22.9 | 4.8 | 5.8 |

**Table S5. $^{13}$C and $^{1}$H chemical shifts of soil and plant samples**. The assignments are identified from $^{13}$C INADEQUATE spectra and $^{1}$H-$^{13}$C HETCOR spectra. The ppm values are shown as $^{13}$C ($^{1}$H), e.g., 88.9 (3.3) represents $^{13}$C ($^{1}$H) chemical shifts (ppm) of cellulose i4. Aromatic rings include guaiacyl (G), syringyl (S), *p*-hydroxyphenyl (H) units. Not applicable (/). Unidentified (-). Uncertain (U).

| **Carbohydrate** | | | | | | | | | | | | | **CO** | Sample |
|---|---|---|---|---|---|---|---|---|---|---|---|---|---|---|
| Cellulose: Interior (i); Surface (s) | | | | | | | Xylan: 2-fold (Xn$^{2f}$); 3-fold (Xn$^{3f}$) | | | | | | | |
| C1 | C2, 3, 5 | iC4 | sC4 | iC6 | sC6 | | C1$^{3f}$ | C4$^{2f}$ | C4$^{3f}$ | C5$^{2f}$ | C5$^{3f}$ | | | |
| 104.9<br>(4.3) | 74.7 (3.4)<br>72.8 (3.5) | 90.2 (3.6)[e]<br>89.5 (3.9)[a,b]<br>87.8 (3.8)[c] | 85.5 (3.5)[h]<br>84.4 (3.5)[f]<br>83.3 (3.5)[g] | 65.9 (3.5)[c]<br>63.7 (3.5)[a,b] | 62.4 (3.6)[f]<br>60.6 (3.6)[g] | | 103.1<br>(4.4) | 81.2<br>(3.6) | 79.3<br>(3.5) | 63.6<br>(-) | 63.2<br>(-) | | 173.4<br>175.0<br>179.6 | #1$^{HF}$ |
| 105.1<br>(4.3) | 75.3 (3.9)<br>74.0 (3.6)<br>72.5 (3.4) | 90.2 (4.0)[e]<br>88.5 (4.8)[a,b]<br>87.6 (5.1)[c] | 86.0 (4.9)[h]<br>83.7 (3.9)[g] | 65.4 (3.4)[c]<br>63.8 (3.7)[a,b] | 62.3 (3.4)[f]<br>61.1 (3.2)[g] | | 102.7<br>(4.2) | 82.0<br>(4.2) | 79.0<br>(4.3) | - | - | | 173.3<br>175.3<br>180.7 | #1$^{Non}$ |
| 104.8<br>(4.3) | 74.6 (3.3)<br>72.8 (3.4) | 89.5 (3.4)[a,b]<br>88.2 (3.7)[c] | 86.7 (3.9)[h]<br>83.9 (3.5)[f,g] | 66.0 (3.8)[c]<br>64.7 (3.5)[a,b] | 63.0 (3.6)[f]<br>61.6 (3.2)[g] | | 103.1<br>(4.4) | 82.7<br>(3.7) | 79.4<br>(3.4) | - | - | | 172.3<br>175.6<br>180.4 | #2$^{HF}$ |
| 105.2<br>(4.4) | 75.7 (3.7)<br>74.6 (3.6)<br>73.3 (3.7) | 89.3 (4.2)[a,b]<br>88.0 (3.9)[c] | 85.7 (3.9)[h]<br>84.3 (4.0)[f]<br>83.2 (3.9)[g] | 65.7 (3.7)[c]<br>64.3 (3.7)[a,b] | 62.7 (3.4)[f] | | 103.3<br>(4.7) | 81.5<br>(4.0) | 77.6<br>(3.7) | - | - | | 174.0<br>175.6<br>180.1 | #4$^{Non}$ |
| 105.0<br>(4.9) | 75.8 (3.5)<br>74.6 (3.7)<br>72.5 (3.5) | 89.4 (4.0)[a,b]<br>87.4 (3.9)[c] | 86.0 (3.7)[h]<br>83.9 (3.8)[f,g] | 66.2 (3.1)[c]<br>64.1 (3.1)[a,b] | 62.3 (3.3)[f] | | 103.4<br>(4.9) | 81.1<br>(4.4) | 79.0<br>(4.3) | - | - | | 173.8<br>176.3<br>180.9 | #6$^{Non}$ |
| 105.0<br>(4.2) | 74.5 (3.3)<br>72.3 (3.3) | 88.8 (3.3)[a,b]<br>87.7 (3.7)[c] | 85.8 (3.7)[h]<br>83.7 (3.3)[f,g] | 64.5 (3.6)[a,b] | 62.4 (3.6)[f] | | 101.9<br>(4.3) | 81.4<br>(3.5) | 78.8<br>(3.5) | 65.2<br>(-) | 63.2<br>(-) | | 174.5 | plant edge |
| 105.0<br>(4.3) | 74.7 (3.3)<br>72.2 (3.4) | 89.0 (3.3)[a,b]<br>87.4 (3.7)[c] | 84.3 (3.3)[f]<br>83.0 (3.4)[g] | 64.6 (3.6)[a,b] | 62.7 (3.4)[f] | | 101.9<br>(4.3) | 81.4<br>(3.5) | 78.9<br>(3.4) | - | 63.6<br>(-) | | 173.1 | plant inland |

| **Aromatic** | | | | | | | | | | | | |
|---|---|---|---|---|---|---|---|---|---|---|---|---|
| S2/6 | G2 | G5, H3/5 | G6 | H2/6 | G1/H1 | S4 | S1 | G4 | G3 | S3/5 | H4 | Sample |
| 105.1<br>(6.0) | 111.5<br>(6.1) | 114.9<br>(6.3) | 117.9 (6.6)<br>121.0 (6.6) | 124.7<br>(6.7) | 128.5<br>(/) | 132.3<br>(/) | 136.5<br>(/) | 145.6<br>(/) | 148.1<br>(/) | 153.6<br>(/) | -<br>(/) | #1$^{HF}$ |
| 104.6<br>(6.0) | 110.1<br>(6.0) | 114.3<br>(6.2) | 118.9 (6.5)<br>122.2 (6.5) | 126.8<br>(5.8) | 129.4<br>(/) | 133.4<br>(/) | 136.4<br>(/) | 145.4<br>(/) | 147.7<br>(/) | 152.6<br>(/) | 162.1<br>(/) | #1$^{Non}$ |
| 105.1<br>(6.1) | 111.3<br>(6.3) | 115.8<br>(6.4) | 117.7 (6.7)<br>121.7 (6.7) | 125.9<br>(6.9) | 129.7<br>(/) | 132.4<br>(/) | 136.1<br>(/) | 143.4<br>(/) | 147.1<br>(/) | 153.4<br>(/) | 163.3<br>(/) | #2$^{HF}$ |
| 106.9<br>(6.2) | 111.1<br>(6.1) | 115.5<br>(6.0) | 118.9 (6.1)<br>122.2 (6.4) | 126.7<br>(6.0) | 130.0<br>(/) | 133.1<br>(/) | 136.8<br>(/) | 143.9<br>(/) | 148.4<br>(/) | 152.5<br>(/) | 159.7<br>(/) | #4$^{Non}$ |
| 105.7<br>(6.1) | 113.0<br>(6.0) | 115.6<br>(6.7) | 118.4 (6.2)<br>122.2 (6.1) | 127.3<br>(6.1) | 130.8<br>(/) | 133.7<br>(/) | 136.8<br>(/) | 144.5<br>(/) | 147.7<br>(/) | 152.9<br>(/) | 161.6<br>(/) | #6$^{Non}$ |
| - | 111.9<br>(6.7) | 116.0<br>(6.6) | 121.6<br>(6.8) | 127.4<br>(6.7) | 130.6<br>(/) | 132.8<br>(/) | 137.0<br>(/) | 144.2<br>(/) | 147.6<br>(/) | 153.0<br>(/) | - | plant edge |
| - | 111.8<br>(6.3) | 116.0<br>(6.5) | 121.7<br>(7.3) | 126.6<br>(6.8) | 131.8<br>(/) | 133.8<br>(/) | - | 145.1<br>(/) | 147.9<br>(/) | 152.8<br>(/) | - | plant inland |

**Aliphatic**

| $-CH_3$ | $-OCO\underline{C}H_3$ | $-\underline{CH_2}-$ CH=CH- | $-CH_2-^b$ | $-CH_2-^a$ | -CCH | $R_3CH$ & $R_4C$ | $-OCH_3^a$ | $-OCH_3^b$ | Sample |
|---|---|---|---|---|---|---|---|---|---|
| 17.9 (0.8) | 23.2 (1.6) | 25.7 (1.4) | 30.6 (1.4) | 33.4 (1.3) | 40.8 (1.8) | 43.6 (2.9) | 50.8 (4.1) | 56.6 (3.5) | #1[HF] |
| 18.7 (0.6) | 23.8 (1.3) | 25.9 (1.6) | 30.1 (1.4) | 33.6 (1.3) | 38.2 (2.8) | 43.3 (3.3) | 50.0 (2.5) | 56.5 (3.3) | #1[Non] |
| 18.0 (0.7) | 23.1 (1.1) | 26.4 (1.1) | 30.1 (1.4) | 33.4 (1.3) | 39.6 (1.8) | 44.3 (3.1) | 50.0 (4.2) | 56.3 (3.6) | #2[HF] |
| 18.5 (0.5) | 22.4 (1.6) | 26.7 (1.3) | 30.2 (1.1) | 34.0 (1.3) | 39.4 (2.8) | 43.5 (2.8) | 50.6 (3.2) | 56.3 (3.2) | #4[Non] |
| 19.5 (0.7) | 23.2 (1.6) | 27.9 (1.1) | 31.0 (1.2) | 34.0 (1.3) | 40.1 (2.4) | 44.6 (2.2) | 51.8 (2.5) | 56.4 (2.9) | #6[Non] |
| - | 22.0 (1.9) | 25.8 (1.4) | 30.2 (1.8) | 32.6 (1.3) | 37.9 (5.2) | - | - | 56.2 (3.5) | plant edge |
| 18.2 (1.0) | 22.1 (1.0) | 26.0 (1.4) | 30.5 (1.3) | 32.8 (1.3) | 38.0 (5.2) | - | - | 58.0 (3.4) | plant inland |

**Table S6. Physiochemical properties of soil samples based on the depth.** Depth is an average value, for example, 5 cm depth means the sample is collected from 0-10 cm section. Samples #1 to #7 are the samples measured by ssNMR and DNP.

| Soil Sample # | Average Depth (cm) | Bulk Density ($g/cm^3$) | Loss on Ignition (Wt %) | Total Carbon (%) |
|---|---|---|---|---|
| **1** | 5 | 0.265 | 20.5 | 8.356 |
| | 15 | 0.240 | 27.1 | |
| | 25 | 0.318 | 17.9 | |
| | 35 | 0.247 | 28.5 | |
| **2** | 45 | 0.294 | 19.9 | 10.826 |
| | 55 | 0.368 | 13.7 | |
| | 65 | 0.141 | 40.0 | |
| | 75 | 0.166 | 43.2 | |
| **3** | 85 | 0.166 | 40.9 | 12.326 |
| | 95 | 0.207 | 31.1 | |
| **4** | 105 | 0.152 | 51.6 | 14.039 |
| | 115 | 0.117 | 59.4 | |
| | 125 | 0.182 | 35.4 | |
| **5** | 135 | 0.114 | 64.8 | 10.717 |
| | 145 | 0.119 | 60.7 | |
| | 155 | 0.146 | 51.6 | |
| **6** | 165 | 0.135 | 39.7 | 11.165 |
| **7** | 175 | 0.306 | 16.1 | 8.114 |

Supplementary References


1 Mentink-Vigier, F. *et al.* Computationally Assisted Design of Polarizing Agents for Dynamic Nuclear Polarization Enhanced NMR: The AsymPol Family. *J. Am. Chem. Soc.* **140**, 11013-11019, doi:10.1021/jacs.8b04911 (2018).
2 Viger-Gravel, J. *et al.* Topology of Pretreated Wood Fibers Using Dynamic Nuclear Polarization. *J. Phys. Chem. C.* **123**, 30407-30415 (2019).
3 Jepsen, R., Roberts, J. & Lick, W. Effects of Bulk Density on Sediment Erosion Rates. *Wat. Air Soil Pollut.* **99**, 21-31 (1997).
4 Craft, C. Freshwater Input Structures Soil Properties, Vertical Accretion, and Nutrient Accumulation of Georgia and U.S Tidal Marshes. *Limnol. Oceanogr.* **52**, 1220-1230 (2007).
5 DeLaune, R. D., Nyman, J. A. & Patrick, W. H. Peat Collapse, Ponding and Wetland Loss in a Rapidly Submerging Coastal Marsh. *J. Coast. Res.* **10**, 1021-1030 (1994).
6 Qu, W. *et al.* Effect of Salinity on the Decomposition of Soil Organic Carbon in a Tidal Wetland. *J. Soils Sediments* **19**, 487-496 (2018).
7 Remusat, L. *et al.* NanoSIMS Study of Organic Matter Associated with Soil Aggregates: Advantages, Limitations, and Combination with STXM. *Environ. Sci. Technol.* **46**, 3943-3949 (2012).
8 Lehmann, J. *et al.* Spatial complexity of soil organic matter forms at nanometre scales. *Nat. Geosci.* **1**, 238-242 (2008).
9 Lattao, C., Birdwell, J., Wang, J. J. & Cook, R. L. Studying Organic Matter Molecular Assemblage within a Whole Organic Soil by Nuclear Magnetic Resonance. *J. Environ. Qual.* **37**, 1501-1509 (2008).
10 Masoom, H. *et al.* Soil Organic Matter in Its Native State: Unravelling the Most Complex Biomaterial on Earth. *Environ. Sci. Technol.* **50**, 1670-1680 (2016).
11 Kelleher, B. P. & Simpson, A. J. Humic Substances in Soils: Are They Really Chemically Distinct? *Environ. Sci. Technol.* **40**, 4605-4611 (2006).
12 Cook, R. L., McIntyre, D. D., Langford, C. H. & Vogel, H. J. A Comprehensive Liquid State Heteronuclear and Multidimensional NMR Study of Laurentian Fulvic Acid. *Environ. Sci. Technol.* **37**, 3935-3944 (2003).
13 Schmidt, M. W. I. *et al.* Persistence of soil organic matter as an ecosystem property. *Nature* **478**, 49-56 (2011).
14 Simon, C. *et al.* Mass Difference Matching Unfolds Hidden Molecular Structures of Dissolved Organic Matter. *Environ. Sci. Technol.* **56**, 11027-11040 (2022).
15 Hertkorn, N. *et al.* Natural Organic Matter and the Event Horizon of Mass Spectrometry. *Anal. Chem.* **80**, 8908-8919 (2008).
